# Molecular basis for a Ca^2+^ binding hierarchy in the EF-hand motifs of CaBP2

**DOI:** 10.1016/j.jbc.2026.113492

**Published:** 2026-08-26

**Authors:** Ryo Motosugi, Erik Walinda, Taehee Kim, Andreea Larisa Mateias, Tatjana Pallinger, Sabin Prajapati, Daichi Morimoto, Myeongkyu Kim, Vladan Rankovic, Christian Griesinger, Kai Tittmann, Tina Pangrsic, Eri Sakata

**Affiliations:** 1 Institute for Auditory Neuroscience, University Medical Center Göttingen, Göttingen, Germany; 2 Multiscale Bioimaging: from Molecular Machines to Networks of Excitable Cells (MBExC), Georg-August University Göttingen, Göttingen, Germany; 3 Department of Molecular and Cellular Physiology, Graduate School of Medicine, Kyoto University, Kyoto, Japan; 4 Institute for Neuropathology, University Medical Center Göttingen, Göttingen, Germany; 5 Collaborative Research Center 1690, University Medical Center Göttingen, Göttingen, Germany; 6 Department for Otolaryngology, Innerearlab, University Medical Center Göttingen, Göttingen, Germany; 7 Department of Molecular Enzymology, Göttingen Center of Molecular Biosciences, Georg-August University Göttingen, Göttingen, Germany; 8 Department of Molecular Engineering, Graduate School of Engineering, Kyoto University, Kyoto, Japan; 9 Department of NMR-Based Structural Biology, Max Planck Institute for Multidisciplinary Sciences, Göttingen, Germany

**Keywords:** calcium-binding protein, calcium channel, calmodulin, nuclear magnetic resonance, electrophysiology, isothermal titration calorimetry, inner hair cells

## Abstract

Calcium-binding protein 2 (CaBP2), a member of the calmodulin (CaM) superfamily, modulates the L-type calcium channel Ca_V_1.3 in the cochlear inner hair cells by suppressing its voltage- and Ca^2+^-dependent inactivation. Like CaM, CaBP2 contains four EF-hand motifs, although only three are functional, yet the precise nature and physiological relevance of its Ca^2+^ binding remain poorly understood. In this study, we present the binding properties of the individual EF-hand motifs of CaBP2L for Ca^2+^ and Mg^2+^ using isothermal titration calorimetry, and nuclear magnetic resonance spectroscopy. The measured dissociation constants of the individual EF-hand motifs span several orders of magnitude, suggesting a hierarchical mode of Ca^2+^ sensing. Our data demonstrate that the CaBP2L structure is stabilized upon Ca^2+^ rather than Mg^2+^ binding. While the N-lobe of CaBP2L significantly contributes to structural fluctuation in the absence of Ca^2+^, this fluctuation is reduced upon Ca^2+^ binding. Furthermore, our electrophysiology experiments show that upon disruption of all three Ca^2+^ binding EF hands of CaBP2 its ability to attenuate Ca^2+^-dependent inactivation is reduced. Therefore, we propose that CaBP2L displays a distinct Ca^2+^ binding property compared to other CaBPs, and that Ca^2+^ binding influences the stability of its structure and consequently channel regulation.

Alterations in cellular calcium ion (Ca^2+^) concentration in synaptic cells trigger various synaptic activities, such as regulation of excitation, synaptic plasticity, and gene expression (1, 2). Entry of Ca^2+^ through voltage-gated calcium channels (Ca_V_) further triggers synaptic transmission. In cochlear inner hair cells (IHCs), approximately 90% of this current is carried through the L-type Ca_V_1.3 channels (3, 4, 5). IHC Ca_V_1.3 channels exhibit rapid gating with a low activation threshold after acoustic stimuli, followed by strikingly slow inactivation (6, 7, 8). Ca_V_1.3 channels (as other members of the Ca_V_ family) undergo inactivation through two distinct pathways, Ca^2+^-dependent inactivation (CDI) and voltage-dependent inactivation (VDI) (9, 10). CDI of Ca_V_1.3 is mediated by calmodulin (CaM), which alters its conformation and interaction with the pore-forming α_1_ subunit of Ca_V_1.3 upon Ca^2+^ binding (9, 11, 12). In contrast, the VDI of Ca_V_1.3 is induced by the movement of four voltage-sensing domains S1–S4, in response to changes in membrane potential, leading to rearrangements in the Ca^2+^ filter and gate formed by the S6 segments (10, 13).

Calcium-binding proteins (CaBPs) are members of the CaM-like superfamily and contain four EF-hand motifs that are helix-loop-helix Ca^2+^ sensors (Fig. 1, *A* and *B*). As in CaM, the four EF-hand motifs of CaBPs are divided into two globular structural domains, the N-lobe (EF1 and EF2) and the C-lobe (EF3 and EF4); however, the EF2 motif of CaBPs is a nonfunctional Ca^2+^ binding motif (14, 15). While the C-lobe is highly conserved, the N-lobe shows significant divergence among CaBPs as well as CaM (Fig. S1). However, the greatest diversity is found in the N-terminal region of CaBPs (N-segment), which is absent in CaM. In several CaBPs, this N-segment contains a single α-helical element (Fig. 1*A*). Similar to CaM, CaBPs interact with L-type Ca_V_ channels, Ca_V_1.2 and Ca_V_1.3, but modulate these channels differently. Notably they inhibit CDI rather than promoting it (16, 17, 18, 19).

**Figure 1:**
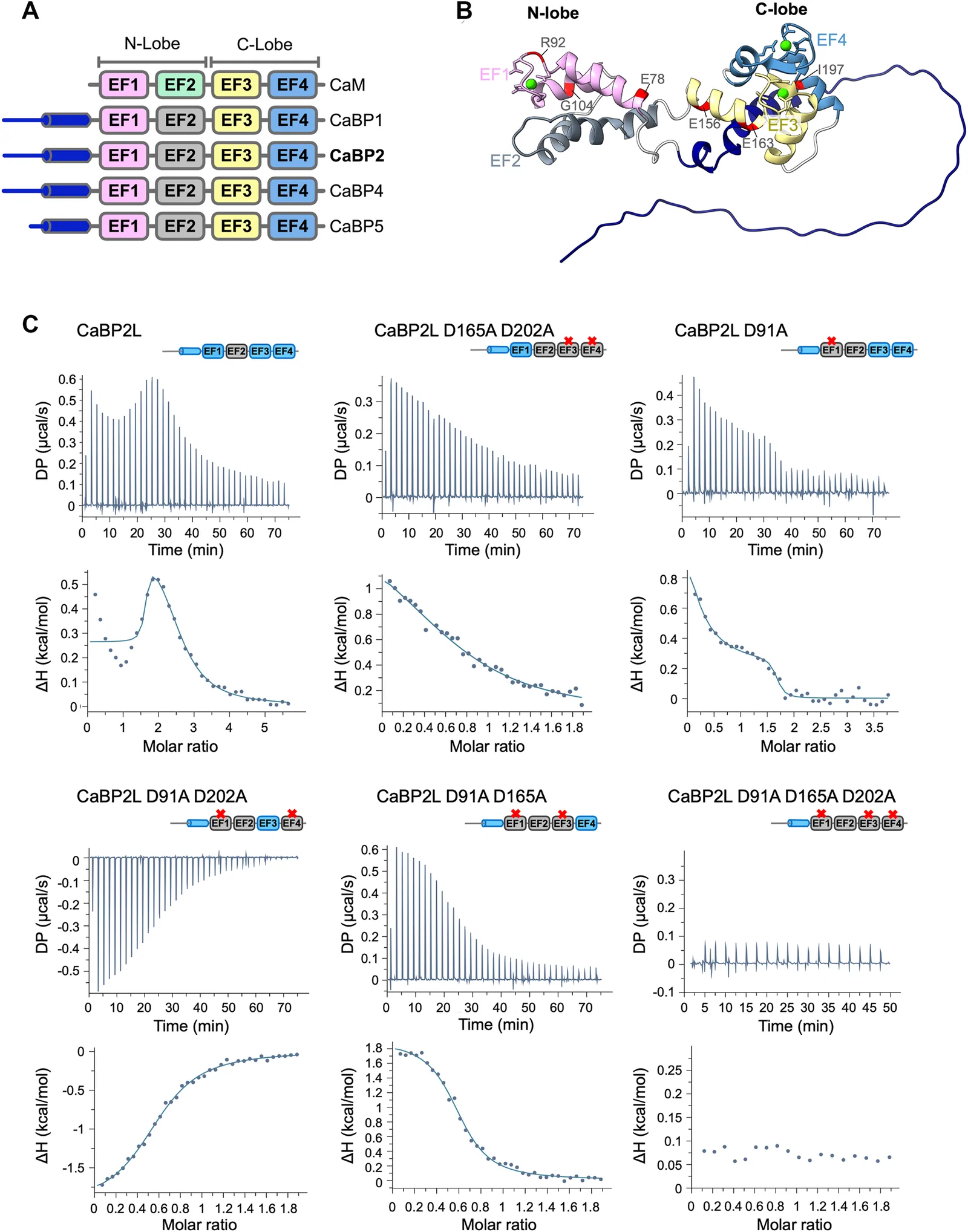
**Isothermal titration calorimetry (ITC) assays of CaBP2L and Ca^2+^-binding mutants.***A*, domain structures of calmodulin (CaM) and CaBPs. Both CaM and CaBPs contain four EF-hand motifs (EF1-4). EF2 of CaBPs (*grey box*) has no Ca^2+^-binding capacity. CaBPs also have an N-terminal segment containing a single helix (shown as a *cylinder*). *B*, structure of CaBP2L predicted by AlphaFold (43). CaBP2L is composed of two globular structural domains, N-lobe (EF1, *pink*; EF2, *gray*) and C-lobe (EF3, *yellow*; EF4, *blue*). The extended N-terminal segment is shown in *navy* and Ca^2+^ ions are shown as *green spheres*. Reported human pathogenic mutations are highlighted in *red* (22). *C*, ITC assays assessing the Ca^2+^-binding affinity of CaBP2L and its mutants. Thermograms (*upper*) and binding isotherms (*lower*) for each measurement are shown. Functional EF-hand motifs in CaBP2L are colored in *light blue*, while disabled EF-hand motifs are shown in *grey*. Mutated motifs are indicated with a *red arrowhead*. Quantitative results are summarized in Table 1.

Among four CaBPs (CaBP1, CaBP2, CaBP4, and CaBP5) expressed in IHCs (17, 20), CaBP2 was identified as a locus for DFNB93 autosomal recessive hearing loss (21, 22, 23) (Fig. 1*B*). In *Cabp2* knockout mice, an increase in Ca_V_1.3 inactivation in IHCs was observed (24), accompanied by reduced accuracy of sound encoding and increased hearing thresholds (24, 25). Unlike several other mouse models for deafness, *Cabp2* knockout mice show normal hair cell (synaptic) development, no hair cell degeneration (24, 25, 26), and so far, no abnormalities in mouse hair cell mechanotransduction have been reported. The canonical CaBP2L variant is the most well-studied CaBP2 isoform, showing attenuation of Ca_V_1.3 CDI in HEK cells as well as partial restoration of hair cell synaptic function and hearing in genetically treated *Cabp2* and *Cabp1/2* knockout mice (24, 27).

Here, we report on the Ca^2+^ binding capability of CaBP2L and its impact on the structure and function to further understand its mechanism of action. Remarkably, CaBP2L displays strong Ca^2+^ binding affinity similar to CaM. Furthermore, NMR analysis shows that truncated CaBP2L lacking the N-segment becomes structurally unstable under low Ca^2+^ concentrations, while the presence of Ca^2+^ ions stabilizes its structure. On the other hand, HEK cell data reveal that mutant CaBP2L with completely lost Ca^2+^ binding is less efficient in attenuating Ca_V_1.3 CDI. Our finding provides significant insights into the molecular properties of CaBP2L and its role in inhibiting Ca_V_1.3 inactivation and buffering Ca^2+^ ions.

## Results

### Ca^2+^-binding affinity of CaBP2L

A previous study reported a relatively modest Ca^2+^ affinity of CaBP2L (CaBP2L from *Homo sapiens*) with *K*_d_ ≈ 50 μM (24), prompting further investigation into its Ca^2+^-sensing capability. First, divalent-metal-ion free (apo) CaBP2L was obtained through protein refolding of denatured CaBP2L to remove residually bound Ca^2+^. Next, the Ca^2+^ affinity of CaBP2L was reassessed by isothermal titration calorimetry (ITC; Fig. 1*C*). The ITC profile exhibited three phases of Ca^2+^ binding; two Ca^2+^ binding phases were observed at lower Ca^2+^ concentrations in addition to the low-affinity site with *K*_d_ ≈ 50 μM (Fig. 1*C*). Due to inter-motif thermodynamic coupling within the lobe, whereby Ca^2+^ occupancy at one EF-hand substantially alters the affinity of its paired EF-hand (discussed further below), binding to the strongest Ca^2+^ binding site (*K*_d_ < 0.12 μM) could not be reliably fitted using the three sets of sites model. We therefore used a two-site model, which allowed us to determine the *K*_*d*_ of the medium Ca^2+^-affinity site (*K*_d_ = 0.120 ± 0.004 μM) and the low Ca^2+^-affinity binding site (*K*_d_ = 40 ± 5 μM) (Table 1). The N-segment deletion mutant CaBP2L_70-220_, referred to as CaBP2LΔNH, shows a slightly reduced Ca^2+^ affinity (Fig. S2*A*). This is consistent with a previous report on the N-segment deletion mutant of CaBP4, showing comparable Ca^2+^ affinity to CaBP2LΔNH (28). Besides, the Ca^2+^-binding affinity of CaBP2S, short isoform of CaBP2 almost completely lacking the N-segment, is also similar to the one of the CaBP2LΔNH, suggesting that the N-segment of CaBP2L stabilizes Ca^2+^ binding (Fig. S2, *A* and *B*, Table S1). Next, we examined Mg^2+^ binding to CaBP2L. ITC analysis revealed an endothermic reaction with remarkably weak affinity (Fig. S2*C*, Table S1), similar to the results obtained with CaM (29, 30).

**Table 1:** ITC thermodynamic parameters for Ca^2+^ binding to CaBP2L and its mutants

Protein	Ligand	K_D_ (μM)	N (sites)	ΔH (kcal/mol)	-TΔS (kcal/mol)
CaBP2L (EF1, EF3, and EF4 active)	Ca^2+^	K_D_ < 0.12	N.D.	N.D.	N.D.
		0.120 ± 0.004	1.40 ± 0.21	0.29 ± 0.06	−9.30 ± 0.17
		40 ± 5	1.20 ± 0.19	0.58 ± 0.03	−6.30 ± 0.47
CaBP2L D165A D202A (EF1 active)	Ca^2+^	49 ± 16	0.63 ± 0.13	1.90 ± 0.77	−7.50 ± 0.57
CaBP2L D91A (C-lobe active)	Ca^2+^	0.19 ± 0.10	0.22 ± 0.08	1.50 ± 0.85	−10 ± 0.87
		1.40 ± 0.88	1.27 ± 0.20	0.27 ± 0.05	−7.90 ± 0.31
CaBP2L D91A D202A (EF3 active)	Ca^2+^	16 ± 4	0.60 ± 0.03	−2.10 ± 0.15	−4.1 ± 0.0
CaBP2L D91A D165A (EF4 active)	Ca^2+^	9 ± 2	0.62 ± 0.03	1.70 ± 0.29	−8.20 ± 0.41

N.D. stands for not determined by ITC. Data are presented as mean ± SD from independent experiments.

### CaBP2L C-lobe exhibits stronger Ca^2+^ affinity compared to the N-lobe

To examine the Ca^2+^ binding affinities of individual EF hand motifs, we purified EF motif mutants in which the conserved first Asp residues at the Ca^2+^ binding sites are replaced with Ala (Fig. S3, *A* and *B*) and performed ITC assays. The triple mutant carrying three D/A mutations (D91A D165A D202A) was not capable of binding Ca^2+^ (Fig. 1*C*). Among the double mutants containing a single active EF-hand motif, only the EF3-active mutant (D91A D202A) exhibited an exothermic response, indicating that the medium-affinity site observed in the wild-type CaBP2L (*K*_*d*_ = 0.12 μM) corresponds to EF3. The affinity measured for the EF1-active mutant (D165A D202A) closely matched the weakest wild-type binding event, indicating that this low-affinity site corresponds to EF1. Remarkably, mutation of a single motif influences the affinities and thermodynamic properties of the remaining EF-hand motifs. In the C-lobe double mutants (EF3-active, D91A D202A; EF4-active, D91A D165A), the observed *K*_d_ values were lower than those in the C-lobe active mutant (D91A) (0.19 μM *versus* 9 μM and 1.4 μM *versus* 16 μM, for EF4 and EF3, respectively). In addition, mutation of the N-lobe (C-lobe active, D91A) markedly reduced the apparent Ca^2+^ affinity of EF3 and EF4 compared to the wild-type, whereas double mutation in the C-lobe (EF1-active, D165A D202A) had only modest effects on EF1 affinity (40 μM *versus* 49 μM), indicating that communication between the lobes is asymmetric. Together, these results provide evidence for cooperative Ca^2+^ binding within the C-lobe and asymmetric inter-lobe communication, whereby perturbation of one Ca^2+^ binding significantly affects Ca^2+^ binding at the others. Consistent with this, previous studies on CaM have shown that mutations at one EF-hand negatively influence the Ca^2+^ affinity of the coupled EF-hand (31, 32). Because the EF-hand motifs are thermodynamically coupled, however, assignment of individual thermodynamic binding events to specific EF-hand motifs remains subject to some degree of ambiguity. Nevertheless, analysis of the double-mutant data and the observed cooperative binding behavior provides a consistent framework for assigning the low-, medium-, and high-affinity Ca^2+^ binding sites to EF1, EF3, and EF4, respectively (Table 1).

We also examined Mg^2+^ binding to the CaBP2L mutants (D165A D202A and D91A). Both mutants bound Mg^2+^ endothermically but with remarkably weak affinity (Fig. S2, *D* and *E*, Table S1). Despite the positive enthalpy change (ΔH > 0), Mg^2+^ binding was driven by a favorable entropy contribution, potentially reflecting the release and increased disorder of water molecules upon complex formation. Although CaBP1 coordinates Mg^2+^ at EF1 through Asp substitution of the conserved Glu at the 12-position on the EF-hand Ca^2+^ binding loop, which enhances specificity for Mg^2+^ ion (33) (see Fig. S1), none of the CaBP2L EF motifs shows Mg^2+^ specificity. Thus, our data demonstrate that individual EF-hand motifs of CaBP2L preferentially bind to Ca^2+^, highlighting its function as a Ca^2+^ sensor that responds to changes in intracellular Ca^2+^ concentration.

### CaBP2L exhibits structural fluctuation in the absence of Ca^2+^

Structures of CaM (34, 35, 36), CaBP1 (33, 37), and CaBP4 (28) were extensively studied by nuclear magnetic resonance (NMR). Two central helices of CaM connecting the N- and C-lobes are reported to switch the conformation from a flexible state (‘closed’ conformation) in the absence of Ca^2+^ (34, 35, 36) to an ‘open’ state by creating an extended helix upon binding to Ca^2+^ (38). Here, a similar approach was adopted to study CaBP2L. Although the ^1^H-^15^N heteronuclear single quantum coherence (HSQC) spectrum of Ca^2+^-CaBP2L showed good chemical shift dispersion, the signal intensities were uneven, with prominent line-broadening observed in the central region of the spectrum (Fig. 2*A*). Deleting the N-segment resulted in remarkably sharpened and well-dispersed peaks in the ^1^H-^15^N HSQC spectrum of CaBP2LΔNH in the presence of saturating Ca^2+^ levels (Fig. 2*A*). In addition, spectral similarities observed between the full length (CaBP2L) and the N-segment truncated construct (CaBP2LΔNH) suggest that the structure of the EF-hand motifs is not largely affected by truncation of the N-terminal segment. Size-exclusion chromatography (SEC) analyses of Ca^2+^-loaded CaBP2L and CaBP2LΔNH revealed the predominant presence of monomeric species, which remained the major population in the apo state. A minor dimeric population was observed only in the apo CaBP2L. Although a dimeric band in the monomer fractions was detected by the non-reducing SDS-PAGE (Fig. 2*B*), its disappearance upon addition of a reducing agent (DTT; Fig. S3*C*) confirms that dimer formation was mediated by transient disulfide bonds. In contrast, dimerization was not observed in the presence of Ca^2+^ (Fig. 2*B*). To further assess the possibility of oligomerization, we performed diffusion NMR experiments on CaBP2LΔNH. The resulting diffusion coefficients are consistent with those expected for a monomer in both the apo and Ca^2+^ bound states and remained unchanged upon doubling the protein concentration from 200 to 400 μM, even at the high protein concentrations required for NMR experiments (Fig. 2*C*).

**Figure 2:**
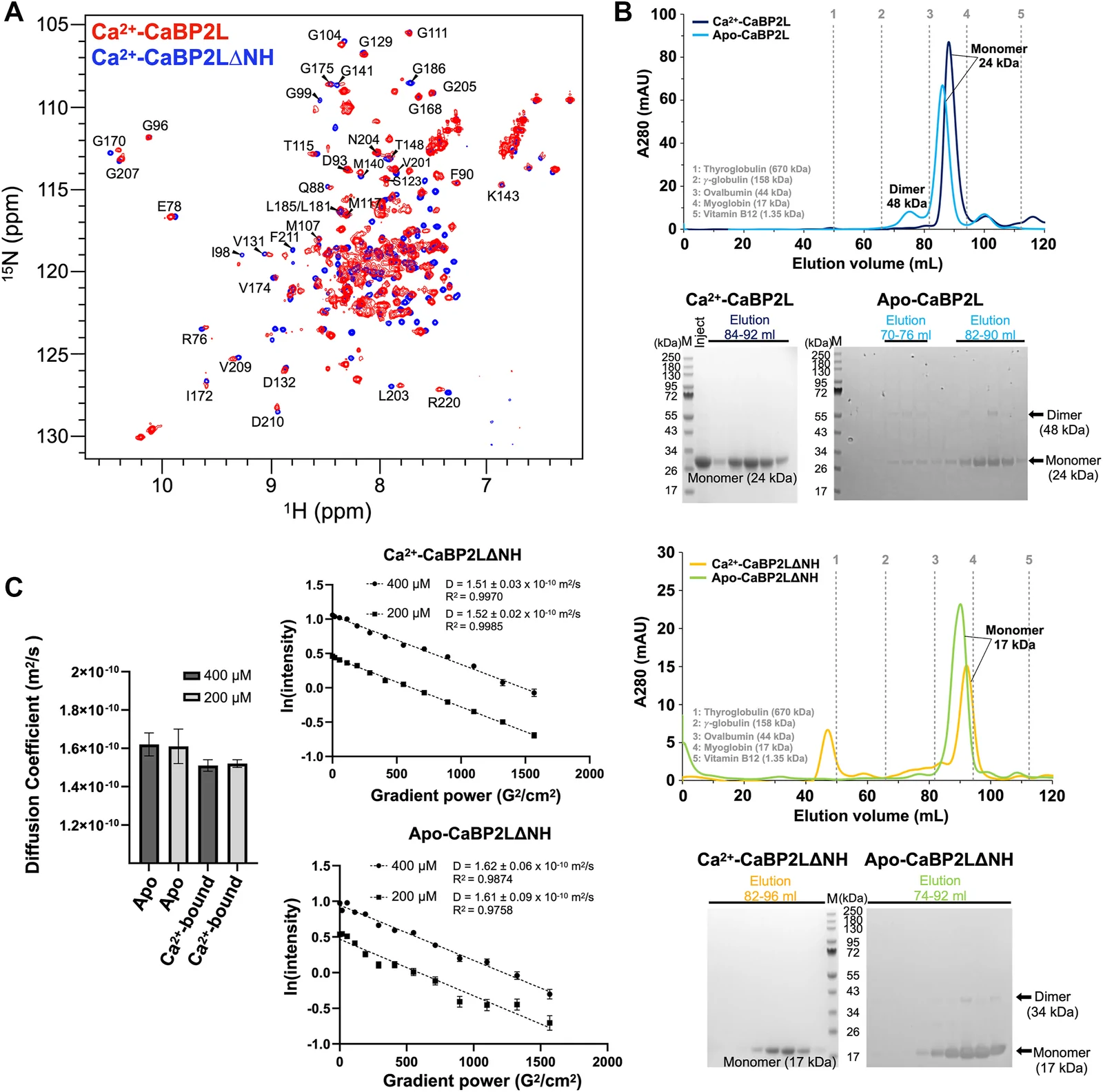
**NMR spectra and size-exclusion chromatography (SEC) analysis of CaBP2L and CaBP2LΔNH.***A*, overlay of ^1^H-^15^N HSQC NMR spectra of the full-length CaBP2L (*red*) and CaBP2LΔNH (*blue*) in the presence of 15 mM of Ca^2+^. Assigned peaks of CaBP2LΔNH in the peripheral region of the spectrum are labeled. *B*, SEC profiles and corresponding SDS-PAGE analyses of full-length CaBP2L (*upper panel*) and CaBP2LΔNH (*lower panel*) in the absence (apo) and presence of Ca^2+^. Because CaBP2L contains five surface-exposed cysteine residues capable of forming intermolecular disulfide bonds, samples were treated with 1 mM TCEP prior to SEC analysis. SEC fractions were subsequently analyzed by SDS-PAGE under denaturing, non-reducing conditions to assess disulfide-linked oligomerization. In the apo state, full-length CaBP2L eluted predominantly as a monomer with a minor dimer peak, whereas CaBP2LΔNH eluted exclusively as a monomer. Nevertheless, SDS-PAGE revealed a small fraction of disulfide-linked dimers within the monomer fractions of both constructs. In the presence of Ca^2+^, only monomeric species were detected by SEC, indicating that the weak dimerization observed is specific to the apo state. *C*, comparison of the diffusion coefficients of apo and Ca^2+^-bound CaBP2LΔNH at different concentrations. Stejskal-Tanner plots of ^1^H diffusion NMR experiments show the natural logarithm of signal intensity as a function of the squared gradient strength (G^2^/cm^2^). The diffusion coefficient (D) was determined from the negative slope of the linear regression. Data represent the mean of all analyzed signals and the error values for the diffusion coefficients were estimated based on the propagation of spectral noise observed within the integrated amide region.

Interestingly, elimination of Ca^2+^ resulted in severe line-broadening of the CaBP2LΔNH spectrum (Figs. 3*A* and S4*A*). Because diffusion NMR data confirmed that apo CaBP2LΔNH remains monomeric, this line-broadening is not due to protein sample aggregation (Fig. 2*C*). Furthermore, the CD spectra of CaBP2L and CaBP2LΔNH demonstrated well-preserved secondary structure in the apo state (Fig. S4*B*), confirming that no protein unfolding took place in the absence of Ca^2+^. The spectrum in the presence of 1 mM Ca^2+^-chelating EGTA showed similarly broadened peaks, demonstrating that the line-broadening did not arise from chemical exchange induced by residual Ca^2+^ ions but rather reflected increased structural dynamics (Fig. 3*A*). In contrast, apo CaM shows only modest peak-broadening and retains sharp peaks in the absence of Ca^2+^ (39), indicating distinct apo-state dynamics. To further characterize these dynamics, we performed transverse relaxation-optimized spectroscopy (TROSY) for rotational correlation time (TRACT) experiments in both the apo and Ca^2+^-bound states (Fig. S4*C*). Ca^2+^-bound CaBP2LΔNH exhibited a slower (τ_c_ = 14.14 ± 0.06 ns) rotational correlation time (τ_c_) compared to its apo state (τ_c_ = 6.10 ± 0.07 ns). A slower Ca^2+^-induced τ_c_ was also observed for CaM, where Ca^2+^ binding alters the anisotropic diffusion tensor and global domain orientation (34, 39). As seen in CaM, Ca^2+^ binding likely stabilizes a more extended conformation, while the apo form adopts a more spherical structure (Fig. S4*C*). Because diffusion NMR showed that CaBP2LΔNH is monomeric in both states, the increased τ_c_ is best explained by Ca^2+^-induced conformational changes rather than oligomerization. Taken together, these results suggest that the Ca^2+^-dependent spectral changes observed for CaBP2LΔNH primarily arise from conformational rearrangements rather than from oligomerization.

**Figure 3:**
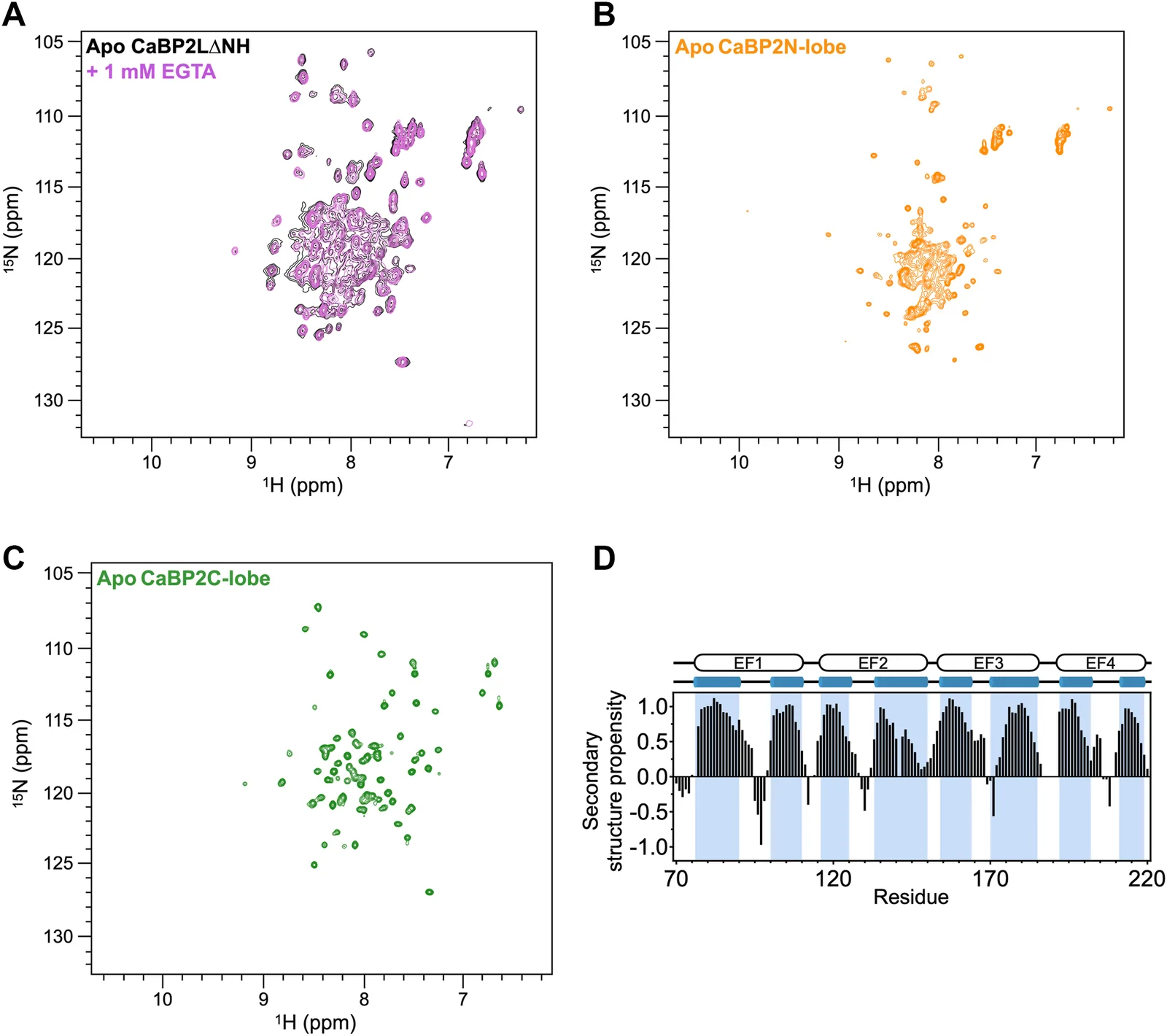
**NMR spectra of CaBP2LΔNH, CaBP2N-lobe, and CaBP2C-lobe in the apo state and secondary structure propensity of Ca^2+^-CaBP2LΔNH.***A*, overlay of ^1^H-^15^N HSQC NMR spectra of the apo CaBP2LΔNH in the absence (*black*) and presence of 1 mM EGTA (*purple*). (B-C) ^1^H-^15^N HSQC NMR spectra of apo CaBP2N-lobe (*B*) and apo CaBP2C-lobe (*C*). *D*, Secondary structure formation propensity of CaBP2LΔNH based on measured Cα and Cβ chemical shifts as calculated by the SSP program (68). At the *top* of the panel, domain structure of EF hand motifs and amino acid residues involved in α-helix formation in a model structure of CaBP2L (obtained from the AlphaFold (43)) are shown schematically; corresponding areas of the figure are shaded in *light blue* for comparison.

In contrast, the addition of Mg^2+^ ions induced chemical shift perturbations, but did not alleviate the severe line broadening, indicating that Mg^2+^ does not sufficiently stabilize the CaBP2LΔNH structure (Fig. S5*A*), consistent with the weak binding observed by ITC (Fig. S2*C*). This is similar to results obtained with Mg^2+^-bound CaM, where the addition of Mg^2+^ also could not induce the tertiary structure changes that Ca^2+^ binding did (40). When Ca^2+^ was titrated into the CaBP2LΔNH in the presence of Mg^2+^, the signals were less sharpened than with Ca^2+^ alone (Fig. S5*A*), suggesting that the presence of Mg^2+^ ion may weaken Ca^2+^ binding. Collectively, the NMR results support our ITC data on preferential binding of CaBP2 to Ca^2+^ and show that Ca^2+^ binding induces structural changes in the protein.

### CaBP2L N-lobe contributes to structural dynamics in apo state

We next investigated the structural modularity of individual lobes of CaBP2L, the N-lobe and the C-lobe, in the absence of divalent metal ions (Fig. 3, *B* and *C*). Whilst the HSQC spectrum of the apo C-lobe indicates a globular structure with dispersed individual resonances, peaks of the apo N-lobe are largely line-broadened, suggesting that the structural fluctuation of CaBP2LΔNH in the apo stateoriginates from the N-lobe in the apo state. Next, Mg^2+^ or Ca^2+^ was titrated into the apo N-lobe and C-lobe. Addition of Mg^2+^ induced chemical shift perturbations in both the N- and C-lobes (Fig. S5, *B* and *C*), but only partially alleviated the line-broadening of the N-lobe. In contrast, provision of Ca^2+^ sharpened the N-lobe signals, indicating that Ca^2+^ rather than Mg^2+^ stabilizes the N-lobe structure in CaBP2L (Fig. S5*B*), as it was previously observed in Troponin C EF-hands (41). CaM C-lobe presents a highly heterogeneous ensemble, while the N-lobe exhibits more restricted dynamics (39, 42). Taken together, our data indicate that CaBP2L displays N-lobe-dominated dynamics, clearly distinguishing it from the behavior described for CaM.

Subsequently, we prepared a ^13^C, ^15^N-double-labeled sample to perform NMR resonance assignment of CaBP2LΔNH (Fig. S6*A*; see Methods section) in the presence of saturating Ca^2+^ concentration. The relatively high concentration of calcium was required to saturate all three functional EF hands with Ca^2+^ to remove residual exchange broadening in the spectra, indicating that at least one of the EF hands had a comparably weak affinity for Ca^2+^ ions, consistent with the ITC data above. The secondary structure calculated based on the assigned chemical shifts of CaBP2LΔNH is consistent with the secondary structure predicted by AlphaFold (43) (Fig. 3*D*).

### Ca^2+^ binding stabilizes the structure of CaBP2LΔNH

Although the apo CaBP2LΔNH contains many low-intensity resonances, we observed the recovery of the intensities upon Ca^2+^ binding. During Ca^2+^ titration, resonances became markedly sharper and additional peaks appeared, indicating stabilization of a specific conformation within the dynamic ensemble upon Ca^2+^ saturation (Fig. S6*B*). Resonances exhibited slow-, intermediate-, and fast-regime chemical exchange on the NMR timescale. Residues undergoing slow- or intermediate-regime exchange could not be assigned in the apo state because of missing peaks. Most residues that appeared only at high Ca^2+^ concentration are located in the C-lobe (Figs. 4*A* and S6*B*). Given that slow-exchange is generally associated with low-micromolar dissociation constants, these observations suggest that the C-lobe binds Ca^2+^ with higher affinity than the N-lobe, which is consistent with our ITC data. Additionally, several peaks that were not detected in the apo state became observable at intermediate Ca^2+^ concentration and exhibited substantial chemical shift perturbations (CSPs) (Fig. S6*B*). Residues in the fast-exchange regime could be reliably tracked throughout the titration and used for CSP analysis (Fig. 4*A*).

**Figure 4:**
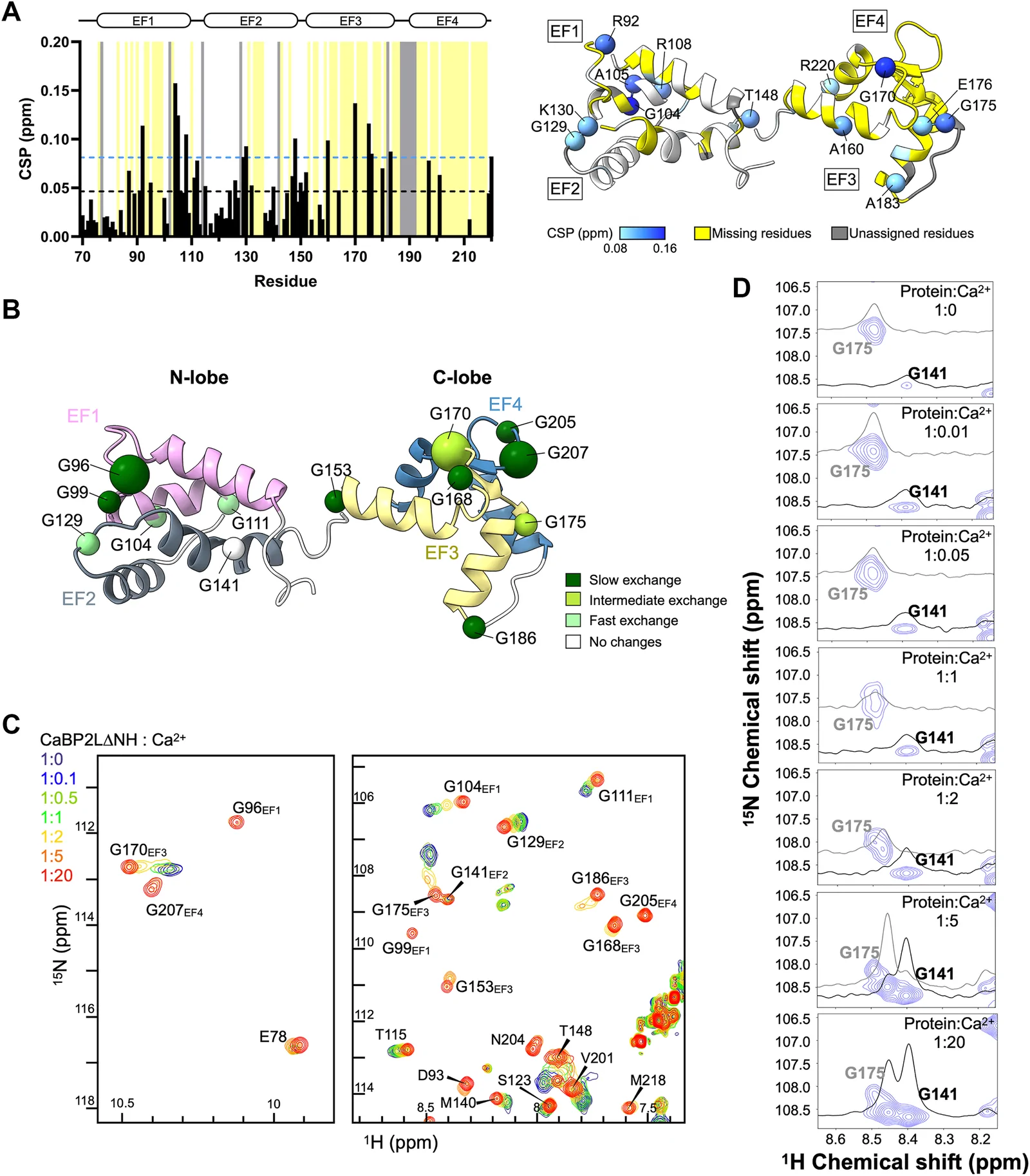
**Ca^2+^ binding of apo CaBP2LΔNH.***A*, (*Left*) Chemical shift perturbation (CSP) values are calculated between the apo state of CaBP2LΔNH (1:0) and the Ca^2+^ saturated state (1:20). CSPs were calculated as: CSP = √((Δ*δ*_*H*_)^2^+(0.1Δ*δ*_*N*_)^2^). The average of CSP is indicated by a *black dashed line*, and the average plus one standard deviation is shown as a *blue dashed line*. Unassigned residues are colored *gray* and residues that are missing in low Ca^2+^ concentrations are shown in *yellow*. (*Right*) CSPs were mapped onto the structural model of CaBP2ΔNH with colors corresponding to the CSP plot. Residues with CSP values above the average plus one standard deviation are represented as spheres. (*B*) Coloring map of Gly residues on structural model of CaBP2LΔNH obtained from AlphaFold (43) at the saturating Ca^2+^ concentration (ratio of 1:20). EF1, 2, 3, and 4 are colored in *pink*, *gray*, *yellow*, and *blue*, respectively, and glycine residues are represented as *spheres*. Conserved Gly residues at the six-position in the EF motifs are shown as *bigger spheres*, while other Gly residues are shown as *smaller spheres*. *Dark green* indicates Gly residues in slow exchange regime; lime indicates residues in intermediate exchange regime; *pale green* indicates residues in the fast exchange regime; and *white* indicates residues showing no changes. *C*, ^1^H-^15^N HSQC spectra of CaBP2LΔNH at different Ca^2+^ concentrations. The colors of the spectra correspond to the colors represented for the molar ratios of CaBP2ΔNH and Ca^2+^ displayed in the figure. Amino acid residues assigned in the Ca^2+^ saturated state (1:20) are also indicated in the figure. *D*, Line-broadening during the titration of CaBP2LΔNH with Ca^2+^. A subregion of the ^1^H-^15^N HSQC spectra highlighting cross-peaks of G141, located near the central helix, and G175, located in the vicinity of EF3, is shown. As the [Ca^2+^] approaches a value where saturation of all binding sites is reached, the linewidth of G141 increasingly sharpens, indicating the stabilization of a well-defined structure at high [Ca^2+^] and structural heterogeneity at low [Ca^2+^]. G175 shows initial line broadening with splitting into individual sub-peaks, whereas later in the titration the resonance sharpens again, indicating [Ca^2+^]-dependent conformational exchange prior to [Ca^2+^] saturation. One-dimensional slices have been taken corresponding to the ^15^N-peak centers in nmrglue (69). Spectra have been normalized for differences in number of scans; differences in protein concentration have not been normalized—the corresponding protein concentrations are from *upper left*.

To further assess Ca^2+^ binding, we focused on the conserved Gly residues at position six of each EF-hand loop (G96, G170, and G207; in EF1, 3 and 4, respectively) (Fig. 4*B*, large spheres). The appearance of downfield-shifted amide chemical shifts (9–10.5 ppm) for these Gly residues is a characteristic hallmark of divalent metal binding in EF-hand proteins (44). At saturating Ca^2+^ concentrations, all three downfield-shifted peaks were observed (Fig. 4*C*). G170 was detected at low Ca^2+^ concentrations and underwent a large CSP upon Ca^2+^ titration, indicating an intermediate-exchange regime. In contrast, G96 and G207 appeared only at high Ca^2+^ concentrations with slow-exchange regime. Even though EF1 has the lowest Ca^2+^ binding affinity, this key Gly residue on the Ca^2+^ binding loop can be stabilized at the low concentration of Ca^2+^. The rest of Gly residues in the C-lobe exhibited slow-exchange (G153, G168, G186, and G205) or intermediate (G175) regime, whereas Gly residues in the N-lobe showed fast-exchange regime behavior (G104, G111, and G129) except residue G99 with slow-exchange regime (Fig. 4, *A*–*C*). Gly175 in EF3 showed peak broadening and recovered the intensity with CSP, while the intensity of Gly141 in EF2 became increasingly sharpened in the Ca^2+^ titration (Fig. 4*D*). Considering that EF2 motif is not able to coordinate Ca^2+^, this change reflects the structural transition to a homogenous conformational ensemble upon Ca^2+^ binding. Interestingly, the CSPs of both Gly170 and Gly175 in EF3 did not follow a linear trajectory from the Ca^2+^-free to the Ca^2+^-saturated states; instead, intermediate states were observed at sub-saturating Ca^2+^ concentrations, likely reflecting allosteric coupling with EF4. Taken together, our Ca^2+^ titration experiments reveal that the binding of Ca^2+^ induces conformational changes in the globular domain of CaBP2L, resulting in increased structural stability.

### Functionality of CaBP2L variants with abrogated Ca^2+^ binding of the EF-hand domains

We next asked whether the efficiency in attenuating Ca_V_1.3 inactivation depends on the ability of the CaBP2L EF-hands to bind divalent ions. For this, we employed a HEK cell line with inducible expression of the Ca_V_1.3 pore-forming α_1D_ subunit and stable expression of Ca_V_ auxiliary subunits β_3_ and α_2_δ-1, as well as *Mm*CaBP2L (CaBP2L from *Mus musculus*) (Fig. 5). Upon induction of α_1D_ expression by tetracycline/doxycycline, the cells displayed variable but sizable depolarization-evoked Ca^2+^ currents, obtained in ruptured patch-clamp recordings (Fig. 5*A*). In the absence of *Mm*CaBP2L, these channels displayed very strong inactivation when tested by 500-ms long depolarizations to the peak Ca^2+^ current potentials (typically −20 mV; Fig. 5, *B*–*D*). This was mostly contributed by CDI as compared to VDI (voltage-dependent inactivation), as the majority of inactivation disappeared upon exchange of Ca^2+^ for Ba^2+^ ions (Fig. 5, *B* and *E*, respectively). Transfection of cells with wild-type *Mm*CaBP2L resulted in comparable peak Ca^2+^ current amplitudes (Fig. 5*A*) but significantly inhibited channel inactivation (Fig. 5, *B*–*D*). To test whether functional *Mm*CaBP2L EF-hands are a prerequisite for the CDI inhibition of Ca_V_1.3 channels, we used two Ca^2+^ binding CaBP2L mutants with Asp to Ala mutations in the EF hand motifs (Fig. S7): the triple mutant with all three functional EF hands disrupted (*Mm*CaBP2L D92A D166A D203A) and a double mutant with disrupted Ca^2+^ binding in the C-lobe (*Mm*CaBP2L D166A D203A). Inactivation in the presence of *Mm*CaBP2L D166A D203A with disrupted C-lobe was functionally indistinguishable from wild-type *Mm*CaBP2L (Fig. 5, *B* and *C*). In contrast, disruption of all three otherwise functional EF hands, *Mm*CaBP2L D92A D166A D203A, resulted in diminished ability to inhibit Ca_V_1.3 CDI. Based on inactivation data alone, we hypothesize that *Mm*CaBP2L with impaired Ca^2+^ binding of all EF hands may not be able to attenuate CDI in the cochlear inner hair cells efficiently.

**Figure 5:**
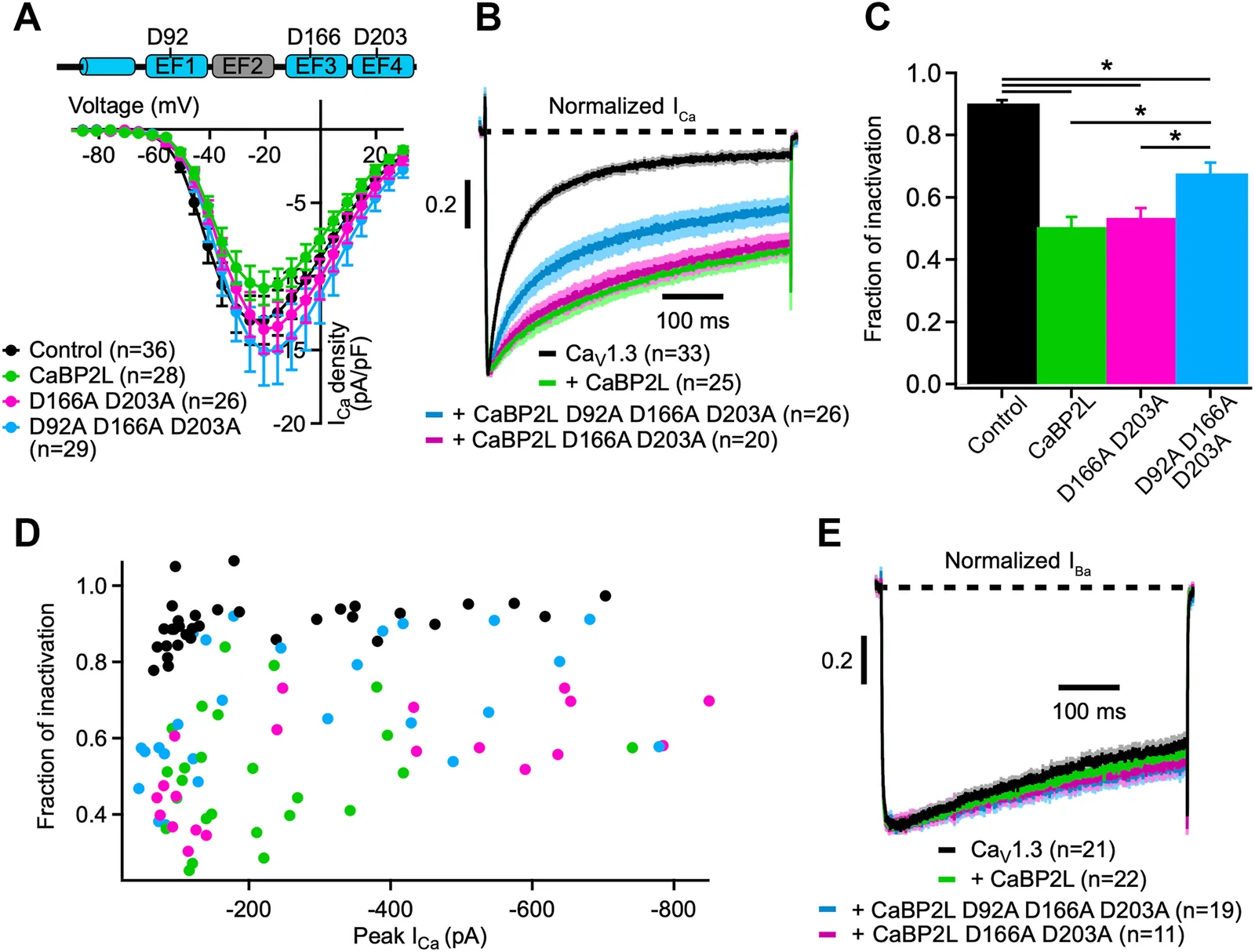
**Ca_V_1.3Ca^2+^current inactivation in the presence of *Mm*CaBP2L with disrupted EF-hand Ca^2+^-binding.***A*, mean current-voltage relationships of HEK cells expressing Ca_V_1.3 in the presence or absence of wild-type *Mm*CaBP2L, or double (D166A D203A) or triple (D92A D166A D203A) *Mm*CaBP2L Ca^2+^-binding mutant (protein scheme depicting the positions of mutations above the IV graph). *B*, peak-normalized mean Ca^2+^-current traces elicited by 500-ms depolarizations to peak Ca^2+^ current potentials. *C*, Fraction of inactivation as measured from residual currents at 500 ms after depolarization start. Note strongly reduced inactivation in the presence of wild-type *Mm*CaBP2L (*green*) and *Mm*CaBP2L with disrupted EF3 and 4 (*magenta*). In the presence of the triple mutant (*blue*), inactivation was stronger as compared to wild-type or double mutant *Mm*CaBP2L (Brown-Forsythe and Welch ANOVA tests: *p* < 0.0001 for control vs wild-type, double and triple mutant *Mm*CaBP2L; *p* < 0.003 for triple vs wild-type; *p* < 0.016 for triple vs double mutant). *D*, Ca^2+^ current inactivation was independent of current amplitudes in the range of the acquired amplitudes. *E*, no effect on VDI is observed in the presence of wild-type or mutant *Mm*CaBP2L, suggesting that the effects on inactivation detected with wild-type *Mm*CaBP2L or mutants can be almost completely attributed to effects on CDI alone. All data is displayed as mean ± SEM. For data dispersion, see *panel D*.

## Discussion

Despite their structural homology to CaM, CaBPs interact with the same L-type Ca_V_ channels but modulate the Ca^2+^ influx differently. CaBP2, as well as CaBP1, are the major CaBPs expressed in the cochlear IHCs (17, 20, 24). In humans, mutations in the *CABP2* gene have been linked to nonsyndromic hearing impairment (21, 23, 45, 46), and the loss of CaBP2 in the mouse KO model results in enhanced inactivation of IHC Ca_V_1.3 channels (24). The lack of CaBP2, alone or in combination with CaBP1, is hypothesized to cause prominent steady-state channel inactivation, thereby reducing availability of channels to trigger synaptic function upon arrival of auditory stimulus (24, 26). In contrast, disruptions of either CaBP1 or CaBP4 alone in mice show only limited effects on IHC Ca_V_1.3 inactivation (17, 25). In this study, we used biophysical and structural approaches to characterize the Ca^2+^ binding of the individual EF-hand motifs of CaBP2L. Unlike the previous report describing a relatively modest Ca^2+^ affinity with *K*_d_ ≈ 50 μM (24), our sample preparation approach through protein refolding enabled precise determination of individual EF-hand affinities for Ca^2+^. The three EF-hand motifs of CaBP2L bind Ca^2+^ ions with varying affinities in a hierarchical fashion, whereas Mg^2+^ was found to interact only weakly with both the N- and C-lobes (Fig. S2, *D* and *E*). Furthermore, deletion of the N-segment in CaBP2L reduces overall Ca^2+^ affinity (Fig. S2*A*), indicating that this region either allosterically increases Ca^2+^ affinity or contributes to stabilization of the EF motifs. Finally, the loss of Ca^2+^ binding from all EF hands diminishes CaBP2L ability to attenuate Ca^2+^ channel inactivation.

We present that three EF hand motifs of CaBP2L bind to Ca^2+^ with varying affinities, enabling it to function as a Ca^2+^ sensor. Although the enthalpic contributions of the binding events could not be accurately resolved due to thermodynamic coupling, the two-site model provided an excellent fit to the Ca^2+^ binding data, supporting the robustness of the affinity estimate. Our ITC and mutagenesis analyses demonstrate that EF4 exhibits the highest Ca^2+^ binding affinity (Table 1), characterized by an endothermic ITC response. In contrast, EF3 exhibits intermediate affinity with an exothermic reaction, whereas EF1 displays the lowest affinity with an endothermic response. Overall, all Ca^2+^ binding reactions are entropy-driven, but only EF3 exhibits a favorable enthalpic contribution (ΔH < 0). Given that a mutation of one EF-hand motif reduces the Ca^2+^ binding affinity of its counterpart within the C-lobe, the two EF-hand motifs mutually stabilize each other. Notably, the N-lobe deficient mutant with active C-lobe (CaBP2L D91A) exhibits a higher affinity compared to the C-lobe deficient mutant with active N-lobe (CaBP2L D165A D202A), which is consistent with higher Ca^2+^ coordination in the CaM C-lobe (33, 34, 47, 48). Cooperative Ca^2+^ binding by EF3 and EF4 leads to a higher overall affinity of CaBP2L C-lobe for Ca^2+^, contributing to its function as a Ca^2+^ sensor and buffering of Ca^2+^ ions. It is worth noting that the C-lobe active mutant (CaBP2L D91A) reduces the Ca^2+^ binding affinity of the EF3 and EF4 motifs by approximately 10-fold compared to the wild type, whereas the EF1 Ca^2+^ binding affinity of the N-lobe active mutant (CaBP2L D165A D202A) exhibits an affinity similar to that of the EF1 obtained in the wild-type CaBP2L (49 ± 16 μM *versus* 40 ± 5 μM for EF1). These results indicate that the individual lobes have different impacts on each other.

In our NMR analysis, line broadening and chemical shift perturbations in the HSQC spectra of CaBP2LΔNH were observed across all three EF-hand motifs, even at low Ca^2+^ concentrations (Fig. 4). Titration experiments supported strong Ca^2+^ binding in the C-lobe with slow exchange kinetics, as their signal intensities were largely recovered at Ca^2+^ saturation. The C-lobes of CaM and CaBPs, with their high Ca^2+^ affinities, are well suited for intercepting Ca^2+^ ions entering through the channels at close to “resting” conditions. Upon cell depolarization, the local Ca^2+^ concentration near the Ca_V_1.3 channel can transiently exceed 100 to 150 μM (49), allowing CaBP2-EF1, 3, and 4 domains to preferably bind Ca^2+^. Together, our study demonstrates that CaBP2L responds to changes in Ca^2+^ concentration using three active EF-hand motifs.

Disruption of metal binding in the CaBP2L C-lobe EF hands showed no detectable effects on HEK cell Ca_V_1.3 Ca^2+^ current inactivation, while simultaneous disruption of all three Ca^2+^ binding sites produced a moderate increase in Ca^2+^ current inactivation (Fig. 5). The difference between the results obtained with the double and triple mutants is surprising, given that the CaBP2L EF1 hand shows the lowest affinity for Ca^2+^. In contrary, disruption of individual or both C-lobe EF hand domains of CaBP1 modestly increased Ca_V_1.2 inactivation, but disruption of EF1 alone had no effect on Ca_V_1.2-mediated Ca^2+^ currents in transiently transfected oocytes (50). Loss of all Ca^2+^ binding sites of CaBP2L increases its conformational heterogeneity, as observed in apo CaBP2LΔNH. This structural fluctuation, which originates from the N-lobe, may affect its interaction with Ca_V_1.3, thereby reducing its ability to attenuate channel inactivation; however, we note that a Ca^2+^-independent effect cannot be completely excluded. Interestingly, such structural fluctuation was not observed in CaBP1 (33). Thus, Ca^2+^ binding of CaBP2 may contribute primarily to structural stability by reducing the conformational heterogeneity rather than directly influencing the modulatory mechanism on Ca_V_1.3. We hypothesize that, in the cochlear IHCs, in the presence of several other channel interaction partners, mutant CaBP2 may be less effective in competing for channel binding. Future experiments in IHCs and animal KO models are required to clarify the behavior of the Ca^2+^ binding mutants and the role of Ca^2+^ binding for the function of CaBP2 and hearing. Furthermore, how the Ca^2+^ binding properties of CaBP2 affect its interaction with the Ca_V_1.3 channel, is currently unknown. Previous reports on CaBP1 and CaBP4 suggest an increased affinity for Ca_V_ IQ domain upon Ca^2+^ binding in CaBPs (28, 50). However, CaBP1 and CaBP4 respond differently to Ca^2+^ and Mg^2+^ ions compared with CaBP2. Moreover, CaBP4 has been reported to engage alternative Ca_V_ interactions, potentially resulting in steric interference or competition with CaM in both apo and Ca^2+^-bound states. (51). Future experiments are required to understand the interactions between the Ca_V_1.3 and CaBP2 and how these are modulated in the presence of CaM and CaBP1.

Recently, we reported that a double knockout of CaBP1 and CaBP2 in mice caused more severe hearing impairment and stronger IHC Ca_V_1.3 inactivation than the lack of CaBP2 alone (27). Disruption of CaBP2 seems to have a significant impact on VDI, rather than on CDI of the IHC Ca^2+^ currents (24), suggesting a strong role of CaBP2 in regulating IHC Ca_V_1.3 VDI and possibly overlapping roles with CaBP1 in attenuating CDI (27). Our findings shed light on the ion-binding specificities of CaBPs, which may underlie the distinct physiological roles of CaBP2 compared to CaBP1. However, further studies are needed to assess the individual roles of CaBP1 and CaBP2 in the IHCs, as well as the exact suppression mechanisms of Ca_V_1.3 CDI and VDI by the two major IHC CaBPs and to understand what role their different metal ion binding properties may have in determining their mechanism of action.

## Experimental procedures

### Plasmids and DNA mutagenesis

The plasmid pET28a encoding human CaBP2L was generated by GenScript. CaBP2L EF-hand mutants in which the conserved Asp in the Ca^2+^-binding loops of individual EF-hand motifs were substituted with Ala were created by using PCR with Phusion High-Fidelity DNA Polymerase (NEB) and primers listed in Table S2. CaBP2L_70-220_ (CaBP2LΔNH), CaBP2L_70-152_ (N-lobe), and CaBP2L_150-220_ (C-lobe) were generated by PCR from the full-length *CABP2* gene using Phusion High-Fidelity DNA Polymerase (NEB). The mutations and corresponding primers are listed (Table S2). After transforming *E. coli* cells (NEB 10-beta) with the PCR products purified using NucleoSpin Gel and PCR Clean-up Kit (MACHEREY-NAGEL), cells were recovered in LB medium at 37 °C for 1 h, and the cell suspension was plated on LB-agar containing kanamycin. The plasmids were then isolated using the NucleoSpin Plasmid Kit (MACHEREY-NAGEL). The plasmid DNA sequences were confirmed by Sanger DNA sequencing (Microsynth Seqlab GmbH).

### Protein expression and purification

Wild-type CaBP2L and its mutants were expressed in *E. coli* BL21(DE3) using a pET28a vector. Expression of the proteins was induced by 1.0 mM isopropyl β-D-1-thiogalactopyranoside (IPTG) at 20 °C in appropriate media. LB medium was used for unlabeled protein and M9 minimal media containing ^15^N-labeled ammonium chloride and [U-^13^C] glucose as the sole nitrogen and carbon sources, respectively, was used for ^15^N-/^13^C-labeled proteins. Cell pellets were lysed in 25 mM HEPES, pH 7.5, 150 mM NaCl, 1 mM DTT, 1 mM phenylmethylsulfonyl fluoride. The lysed cells were sonicated for 5 min in ice at 40% amplitude (0.5 s on and 0.5 s off cycle) using the Branson Sonifier (Branson Ultrasonics Corporation). After clarification of the cell lysate by centrifugation (4 °C, 47.000 *g*), the supernatant was loaded onto a Ni^2+^ Sepharose 6 Fast Flow column (Cytiva). CaBP2L protein was washed with lysis buffer and denatured with 8.0 M urea on the column. Ca^2+^ ions were removed by applying 20 column volumes of 8.0 M urea-containing buffer. The denatured CaBP2L was then eluted by imidazole in the presence of 8.0 M urea and the eluted fraction was slowly diluted in a refolding buffer containing 25 mM HEPES, pH 7.5, 150 mM NaCl, 6 mM L-glutathione reduced (GSH, ITW Reagents), and 0.6 mM L-glutathione oxidized (GSSG, Carl Roth), as previously described (52). Refolded protein was concentrated on the Ni^2+^ Sepharose 6 Fast Flow. After imidazole elution, the His-tag of the proteins was cleaved by thrombin protease (Cytiva). Subsequently, CaBP2L was purified by size-exclusion chromatography on a HiLoad 16/600 Superdex column (Cytiva) in a buffer consisting of 25 mM HEPES, pH 7.5 and 150 mM NaCl. Purified apo-CaBP2L was dialyzed twice against Chelex 100-treated buffer (0.8%) to remove residual Ca^2+^. Protein purity was confirmed by SDS-PAGE, and concentrations were determined by UV-VIS spectrophotometry. Mutants of CaBP2L were expressed and purified as described for the wild-type protein.

### Multiple sequence alignment

Sequence alignment was produced by Clustal Omega (53) and depicted using ESPript 3.2 (54). The UniProt accession numbers of the proteins are as follows: Q9NPB3-1 for human CaBP2L, Q9NPB3-2 for human CaBP2S, Q9NZU7-1 for human CaBP1L, P0DP23 for human CaM, and Q9JLK4 (NP_038906.2) for mouse CaBP2L. The predicted secondary structure of the CaBP2L was obtained from AlphaFold prediction (AF-Q9NPB3-F1) (43).

### Isothermal titration calorimetry

CaBP2L and its mutants were concentrated to 200 to 250 μM and degassed for 10 min before the ITC experiments. A total of 37 injections (1 μl) of CaCl_2_ were made at concentrations 10, 20, or 30 times higher than the protein sample. The protein and ligand concentrations used in each experiment are specified in Table S3. Control titration was performed using a buffer blank to account for the heat of dilution and no effects were observed. Background subtraction was performed on each experiment by MicroCal PEAQ-ITC analysis software; however, the subtraction between different experiments was not performed. The results were analyzed using MicroCal PEAQ-ITC analysis software, and after background subtraction, the thermodynamic coefficients were also calculated. All the ITC experiments were performed at 8 °C.

### Circular dichroism spectroscopy

Apo CaBP2L and Apo CaBP2LΔNH (0.08 mg/ml) were prepared in a buffer containing 25 mM HEPES (pH 7.5) and 150 mM NaCl. Chirascan Circular Dichroism Spectrometer (Applied Photo physics Ltd) was used to analyze the secondary structure of CaBP2L and CaBP2LΔNH. The spectral range was 180 to 260 nm with a step of 1 nm and a bandwidth of 1 nm. All samples were measured 10 times, and the average was calculated. All the measurements were performed at 20 °C.

### Size-exclusion chromatography analysis

Because CaBP2L contains five surface-exposed cysteine residues capable of forming intermolecular disulfide bonds, purified CaBP2L (1 mg/ml) or CaBP2LΔNH (1 mg/ml) was treated with 1 mM TCEP prior to SEC analysis. The samples were applied to HiLoad 16/600 Superdex column (Cytiva) gel filtration column equilibrated in gel filtration buffer containing 25 mM HEPES, pH 7.5, 150 mM NaCl, 1 mM tris(2-carboxyethyl)phosphine (TCEP), and 0 or 1 mM CaCl_2_. The eluted protein was detected by monitoring absorbance at 280 nm. For calibration, Gel Filtration Standard (Bio-Rad, #1511901) was used. After fractionation, SDS-PAGE was performed under denaturing but non-reducing conditions to assess potential oligomer formation through disulfide bonds.

### NMR spectroscopy

NMR experiments for the assignment and Ca^2+^ titration of CaBP2LΔNH were performed on a Bruker Avance II spectrometer (^1^H Larmor frequency: 700 MHz) equipped with a 5 mm ^15^N/^13^C/^1^H *z*-gradient triple resonance cryoprobe (Bruker BioSpin). All NMR measurements were performed at 298 K.

Prior to the NMR measurements for the assignment, CaBP2LΔNH was buffer exchanged into NMR buffer [25 mM HEPES pH 7.5, 150 mM NaCl, 15 mM CaCl_2_, 1 mM Tris(2-carboxyethyl)phosphine (TCEP, Nacalai Tesque), 4% deuterium oxide, 0.2 mM DSS (sodium 2,2-dimethyl-2-silapentane-5-sulfonate, Tokyo Chemical Industry)]. The sample was then placed in Shigemi tubes at a final sample volume of 300 μl at a concentration of 0.6 mM. To identify amide cross-peaks not observed at pH 7.5, the sample was dialyzed against a lower pH buffer (20 mM piperazine-N,N′-bis(2-ethanesulfonic acid) (PIPES, Dojindo) pH 6.2, 1 mM TCEP, 15 mM CaCl_2_). Figures were drawn by NMRDraw (55) and annotated in Inkscape.

For the Ca^2+^ titration of CaBP2LΔNH, ^1^H-^15^N HSQC spectra were acquired with spectral widths of 12 × 28 ppm (^1^H × ^15^N), 1024 × 256 complex points, and 4 scans (0 eq. and 20 eq.) or 8 scans (0.1, 0.5, 1, 2, and 5 eq.), requiring approximately 40 and 80 min, respectively, for measurement. All the apo-state NMR samples were prepared in buffer containing 25 mM HEPES, pH 7.5, 150 mM NaCl and 10% D_2_O and were placed in 3 mm tubes at a final sample volume of 200 μl. The Apo CaBP2LΔNH sample was prepared at a concentration of 0.4 mM in the absence and presence of 1 mM EGTA. To obtain the NMR spectra in the presence of Mg^2+^ and Ca^2+^, the sample was supplemented with 1.5 mM MgCl_2_ and then was supplemented with 8 mM CaCl_2_. The Apo CaBP2 N-lobe/C-lobe samples were prepared at a concentration of 0.2 mM. The samples were supplemented with 1 mM MgCl_2_ or 10 mM CaCl_2_.

All NMR spectra of apo-state samples (Apo CaBP2LΔNH, Apo CaBP2 N-lobe and Apo CaBP2 C-lobe) were recorded on a Bruker 700 MHz HD Avance III spectrometer equipped with 5 mm ^1^H-^13^C/^15^N/D CP TCI cryoprobe. 2D ^1^H-^15^N HSQC spectra of apo CaBP2LΔNH were acquired with spectral widths of 12 × 30 ppm (^1^H × ^15^N), 1024 × 256 complex points, and 8 scans for 55 min. 2D ^1^H-^15^N HSQC spectra of apo CaBP2 N-lobe were acquired with spectral widths of 12.3 × 30 ppm (^1^H × ^15^N), 1024 × 256 complex points, and 16 scans for 75 min 2D ^1^H-^15^N. HSQC spectra of apo CaBP2 C-lobe were acquired with spectral widths of 14 × 30 ppm (^1^H × ^15^N), 1024 × 256 complex points, and 8 scans for 55 min. All 2D datasets were processed in NMRPipe (55) and analyzed in NMRFAM-Sparky (56) and CcpNMR Analysis (CCPN version 3.2.0) (57).

### Diffusion NMR and TRACT experiments

All diffusion NMR and TRACT (TROSY for rotational correlation times) spectra of apo and Ca^2+^ bound CaBP2LΔNH were recorded at 298 K on a 600 MHz Bruker Avance NEO spectrometer equipped with 5 mm ^1^H&^19^F-^13^C/^15^N-D CPP TCI cryoprobe with *z*-gradient. For diffusion NMR experiments, spectra were acquired with a diffusion time (Δ) of 75 ms and a pulsed gradient duration (δ) of 3.0 ms. Gradient strength was incremented in 13 linear steps from 5 to 95% of the maximum gradient strength (47.0 G/cm for a smoothed square gradient pulse). The spectra were processed with the software Topspin 4.5.0 (Bruker). The translational diffusion coefficient (D) was determined by fitting the linearized diffusion data to the Stejskal-Tanner equation (58, 59):

$$\ln\left(I\right)=-D\cdot K\cdot G^{2}+\ln\left(I_{0}\right)$$

where I is the signal intensity, G is the gradient strength, and K is the constant defined by the pulse sequence parameters $\gamma^{2}\delta^{2}\left(\Delta -\delta /3\right)$. The value of D was derived from the negative slope of the linear regression of ln(I) *versus* $G^{2}$ ($G^{2}/{cm}^{2}$). Here, γ represents the gyromagnetic ratio of ^1^H.

For TRACT experiment (60, 61), spectra were acquired with 0.2 mM concentration of apo and Ca^2+^ bound CaBP2LΔNH. For the ^13^C/^15^N-labeled apo-CaBP2LΔNH, the TRACT pulse sequence was supplemented with ^13^C refocusing pulses and an explicit ^13^C purge block. These modifications were implemented to refocus one-bond ^1^J_CN_ couplings and eliminate residual ^13^C magnetization, thereby ensuring ^15^N relaxation measurements remained unaffected by ^13^C interference. The Ca^2+^-bound CaBP2LΔNH was analyzed using a ^15^N-labeled sample with a standard TRACT pulse sequence, omitting ^13^C-specific elements. Relaxation rates for the $R_{2\alpha }$ and $R_{2\beta }$ components of the ^15^N doublet were extracted from the negative slope of linear regressions of ln(I) *versus* relaxation delay time. The rotational correlation time, τ_c_, was estimated from the cross-correlated relaxation rate difference ($\Delta R_{2}=R_{2\beta }-R_{2\alpha }$) using the relation $\tau_{c}\approx \Delta R_{2}/1.5$.

Signal intensities were integrated across the amide proton region between 7.5 and 8.5 ppm. Experimental uncertainties were estimated using the root-mean-square deviation (RMSD) of a baseline region of equivalent width.

### Assignments and data deposition

For backbone and partial sidechain assignment, the following experiments were recorded: ^1^H-^15^N HSQC, ^1^H-^13^C HSQC, HNCO, HN(CA)CO, HNCA, HN(CO)CA, CBCA(CO)NH, HNCACB, HBHA(CO)NH, HBHANH, H(CCO)NH, C(CO)NH, ^1^H-^13^C NOESY-HSQC (aliphatic region), and ^1^H-^15^N NOESY-HSQC (62). The resultant NMR data were then processed in NMRPipe (55) with direct (^1^H) and indirect (^13^C, ^15^N) referencing of chemical shifts with respect to DSS as described before (63, 64), and displayed in CcpNmr analysis version 2.5 (57, 65). Peak lists for each experiment were obtained by manual peak picking from the respective HSQC root resonances in CcpNmr analysis; these lists were then exported in XEASY format for use in FLYA (66). The plasmid-derived N-terminal sequence (Gly)-Ser-His was excluded from the assignment process. FLYA obtained reliable resonance assignments for most of the protein sequence, as confirmed by manually checking the FLYA-predicted assignments in CcpNmr analysis (exclusively manually confirmed resonances are reported herein). Of the 149 expected backbone amide signals (excluding three proline residues and the plasmid-derived N-terminal tag residues Gly-Ser-His), 141 were assigned, corresponding to 94.6% completeness; the eight unassigned amide signals are detailed below. The following amide HSQC cross-peaks remained unidentified: Arg101, Glu102, Gly128, Lys182, as well as several residues in the linker between EF3 and EF4, likely owing to either conformational flexibility in that region or hydrogen exchange. Interestingly, although Arg101 and Gly128 were not observed at pH 7.5 — likely due to hydrogen exchange at higher pH — these residues could be identified in a confirmatory dataset collected at pH 6.2 (comprising a ^1^H-^15^N HSQC, CBCA(CO)NH, and HNCACB); in addition, the EF3-EF4 linker contains a couple of unassigned resonances in another reported CaBP protein (67), similar to our observation for CaBP2LΔNH. For several residues in the N-terminal region two distinct amide resonances with similar intensities were observed; these are indicated by subscript “m” in the Figure S6*A* and might arise from either cis/trans isomerization of the distal Pro residue (Pro77) as this region is unstructured or a different kind of conformational exchange. As previously reported for CaBP1 (67), we also observed three downfield shifted Gly amide proton resonances in the vicinity of 10.5 ppm. In the case of CaBP2LΔNH, these Gly residues corresponded to members of EF motifs 1 (Gly96), 3 (Gly170), and 4 (Gly207), consistent with the idea that EF1, EF3, and EF4 are in a Ca^2+^ ion-bound state (33). Typical acquisition parameters for the triple-resonance experiments were as follows: for all experiments, the ^1^H direct dimension was acquired with 1024 complex points. For the carbonyl experiments (HNCO, HN(CA)CO), spectral widths were 10 × 27 × 7 ppm (^1^H × ^15^N × ^13^C) with 1024 × 64 × 64 complex points and 16 scans per increment; for the C_α_ experiments (HNCA, HN(CO)CA), 14 × 35 × 28 ppm with 1024 × 48 × 64 points and 16 scans per increment; and for the C_α_/C_β_ experiments, 14 × 35 × 64 ppm with 1024 × 48 × 80 points and 32 scans (CBCA(CO)NH) or 64 scans (HNCACB) per increment, with the latter two requiring approximately 2 to 3 days each. The ^1^H-^15^N HSQC was acquired with spectral widths of 12 × 28 ppm, 1024 × 512 complex points, and 4 scans per increment. We have deposited our assignments including backbone and partial sidechain (H^α^, H^β,^ H^γ^, C^β^) in the BioMagResBank under accession number 26338.

### HEK293 cell culture

Human embryonic kidney (HEK) 293 cells with tetracycline-inducible expression of Ca_V_1.3 pore-forming α_1D_ subunit and constitutive expression of the auxiliary β_3_ and α_2_δ1 subunits were purchased from Charles River Laboratories (#CT6232 Human Ca_V_1.3/β3/α2δ1-HEK cell line, Cleveland, USA) and were used according to the manufacturers protocol. The cells were cultured in the Dulbecco’s modified Eagle’s medium (Life Technologies, Paisley, UK) with 10% FBS (Life Technologies), 0.6 μM isradipine or 3 μM verapamil (Sigma-Aldrich; both L-type Ca_V_ channel blockers) and selection antibiotics (Penicillin-Streptomycin, Blasticidin, Geneticin, Zeocin, Hygromycin) at 37 °C in 5% CO_2_. For the induction of Ca_V_1.3 channel expression 3 μg/ml tetracycline or doxycycline were added to the selection antibiotic-free media containing Ca^2+^ channel blocker. 24 to 48 h after induction the cells were well attached and appeared healthy. At 70 to 80% confluence, the cells were transfected with 150 to 500 ng/μl cDNAs encoding mouse *CaBP2-L*: wild-type CaBP2L (NCBI Reference Sequence: NP_038906.2), or Ca^2+^-binding mutants CaBP2L D92A D166A D203A (triple mutant) and D166A D203A (double mutant). A P2A peptide sequence was implemented for bicistronic expression of the CaBP2 and enhanced green protein (eGFP). The plasmids contained a hybrid hCMV/hBA (human beta actin) promoter to support strong transgene expression, the transcript the woodchuck hepatitis virus posttranslational regulatory element (WPRE), and the bovine growth hormone (bGH) polyadenylation sequence for enhanced expression and better stability, and additional regulatory domains as the same construct was then packaged into viral capsids to enable comparable experiments with future *in vivo* injections in mice. For transfection of HEK cells Lipofectamine (Invitrogen) was used according to the manufacturer’s protocol. Cells treated with the transfection mixture were incubated at 37 °C for 24 h, plated on 12 mm dishes, and used in whole-cell patch-clamp recordings 28 to 48 h after transfection.

### Patch-clamp of HEK cells

Whole-cell ruptured patch-clamp recordings were performed at room temperature with an EPC-9 and EPC-10 patch-clamp amplifier in combination with the Pulse/Patchmaster software (HEKA Elektronik, Lambrecht, Germany). The intracellular solution contained in 140 mM NMDG, 10 mM NaCl, 10 mM HEPES, 5 mM EGTA, 3 mM Mg-ATP and 1 mM MgCl_2_. pH was adjusted to 7.3 with Methansulfonic acid and Osmolarity to about 290 mosmol/l with NMDG. The extracellular solution contained: 150 mM Choline-Cl, 10 mM HEPES, 1 mM MgCl_2_ and 10 mM CaCl_2_. In a subset of experiments CaCl_2_ was replaced by an equal concentration of BaCl_2_ to isolate the voltage-dependent component of channel inactivation. Both extracellular solutions were adjusted to a pH of 7.3 with CsOH and the osmolarity to 300 to 310 mosmol/l with Choline-Cl. Pipette resistances were typically approximately 5 MΩ in the bath solution. Leak subtraction was conducted using a P/10 protocol. All voltages were corrected for liquid junction potential (−20 mV). The cells were visualized under Olympus BX50WI microscope (Olympus). Successful transfection was verified using Till Photonics imaging system or LedHUB (Omicron) with Andor Zyla4.2 camera to detect fluorescence in the cells expressing CaBP2 and GFP. To measure Ca^2+^ current density, Ca^2+^ currents (*I*_Ca_) were evoked by 10-ms pulses from −90 mV to various potentials and normalized to the cell membrane capacitance. We selected cells with the peak current amplitude over 60 and below 900 pA for further experiments. To measure Ca^2+^ channel inactivation, currents were evoked by 500-ms long depolarizations to the peak Ca^2+^ current potential (typically −20 mV). Fractional *I*_Ca_ was defined as the residual current amplitude at the end of the pulse normalized to the peak current amplitude.

### Data and statistical analysis

Patch-clamp data were analysed using Igor Pro (Wavemetrics, Portland, OR, USA). Graphs were created by using Igor Pro. Statistical analysis was performed using Igor Pro and GraphPad Prism. Significant differences were determined by Welch ANOVA test. Data were incorporated into figures using Igor Pro, and Affinity Designer software. All averaged data represent the mean ± SEM from several transfections. For every dataset the number of cells (n) is indicated.

## Data availability

We have deposited our assignments of human CaBP2LΔNH (70–220) including backbone and partial sidechain (Hα, Hβ, Hγ, Cβ) in the BioMagResBank under accession number 26338. Additional NMR data are available upon reasonable request to the authors.

## Supporting information

This article contains supporting information.

## Conflict of interest

The authors declare that they have no conflicts of interest with the contents of this article.
