# Supplementary material for: Molecular basis for a Ca2+ binding hierarchy in the EF-hand motifs of CaBP2
Source: J Biol Chem. 2026 Aug 26;302(10):113492. doi: 10.1016/j.jbc.2026.113492 (PMC13659490; doi:10.1016/j.jbc.2026.113492)
Supplement: Supplementary file 1 [file mmc1.pdf]

Supporting information

Molecular basis for a Ca^{2+} binding hierarchy in the EF-hand motifs of CaBP2

Ryo Motosugi[1,2,*], Erik Walinda[4,*], Taehee Kim[1,2,3,9*], Andreea Larisa Mateias[5,9], Tatjana Pallinger[5], Sabin Prajapati[6], Daichi Morimoto[7], Myeongkyu Kim[2,8], Vladan Rankovic[1], Christian Griesinger[2,8], Kai Tittmann[6], Tina Pangrsic[1,2,5,9], Eri Sakata[1,2,9]

[1] Institute for Auditory Neuroscience, InnerEarLab, University Medical Center Göttingen, Göttingen, Germany
[2] Multiscale Bioimaging: from Molecular Machines to Networks of Excitable Cells (MBExC), Georg-August University Göttingen, Göttingen, Germany
[3] Institute for Neuropathology, University Medical Center Göttingen, Göttingen, Germany
[4] Department of Molecular and Cellular Physiology, Graduate School of Medicine, Kyoto University, Kyoto, Japan
[5] Department for Otolaryngology, Innerearlab, University Medical Center Göttingen, Göttingen, Germany
[6] Department of Molecular Enzymology, Göttingen Center of Molecular Biosciences, Georg-August University Göttingen, Göttingen, Germany
[7] Department of Molecular Engineering, Graduate School of Engineering, Kyoto University, Kyoto, Japan
[8] Department of NMR-Based Structural Biology, Max Planck Institute (MPI) for Multidisciplinary Sciences, Göttingen, Germany
[9] Collaborative Research Center 1690, University Medical Center Göttingen, Göttingen, Germany

* These authors contributed equally.

To whom correspondence shall be addressed: Eri Sakata: eri.sakata@med.uni-goettingen.de, Tina Pangrsic: tpangrs@gwdg.de, Erik Walinda: walinda.erik.6e@kyoto-u.ac.jp

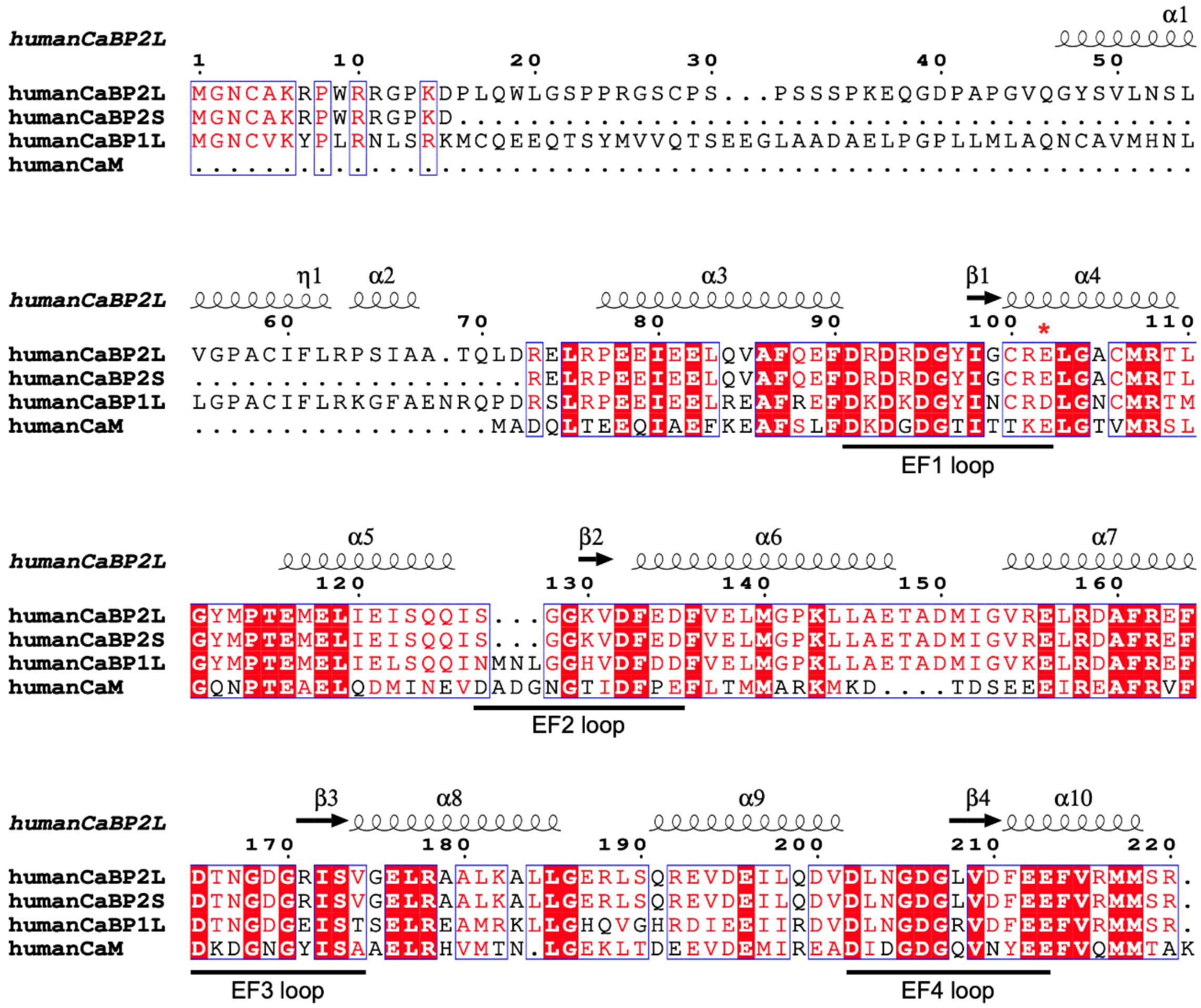

Figure S1 Multiple sequence alignment of CaBP2L, CaBP2S, CaBP1L, and CaM. The sequence of the CaBPs EF2 motif differs from CaM. Both CaBP2L and CaBP1L contain an extended N-terminal segment. The multiple sequence alignment was produced by Clustal Omega (53) and drawn using ESPript 3.2 (54). The UniProt accession numbers of the proteins are as follows: Q9NPB3-1 for human CaBP2L, Q9NPB3-2 for human CaBP2S, Q9NZU7-1 for human CaBP1L, and P0DP23 for human CaM. The predicted secondary structure of the CaBP2L obtained from AlphaFold (43) (AF-Q9NPB3-F1) are shown on top of the alignment: α, α-helices; β, β-strands; η, 3_{10}-helix. The EF hand Ca^{2+}-binding loops are underlined below the sequence of human CaM. The asterisk in red indicates the residue in the EF1 hand Ca^{2+}-binding loop of CaBP1 where Glu is replaced with Asp.

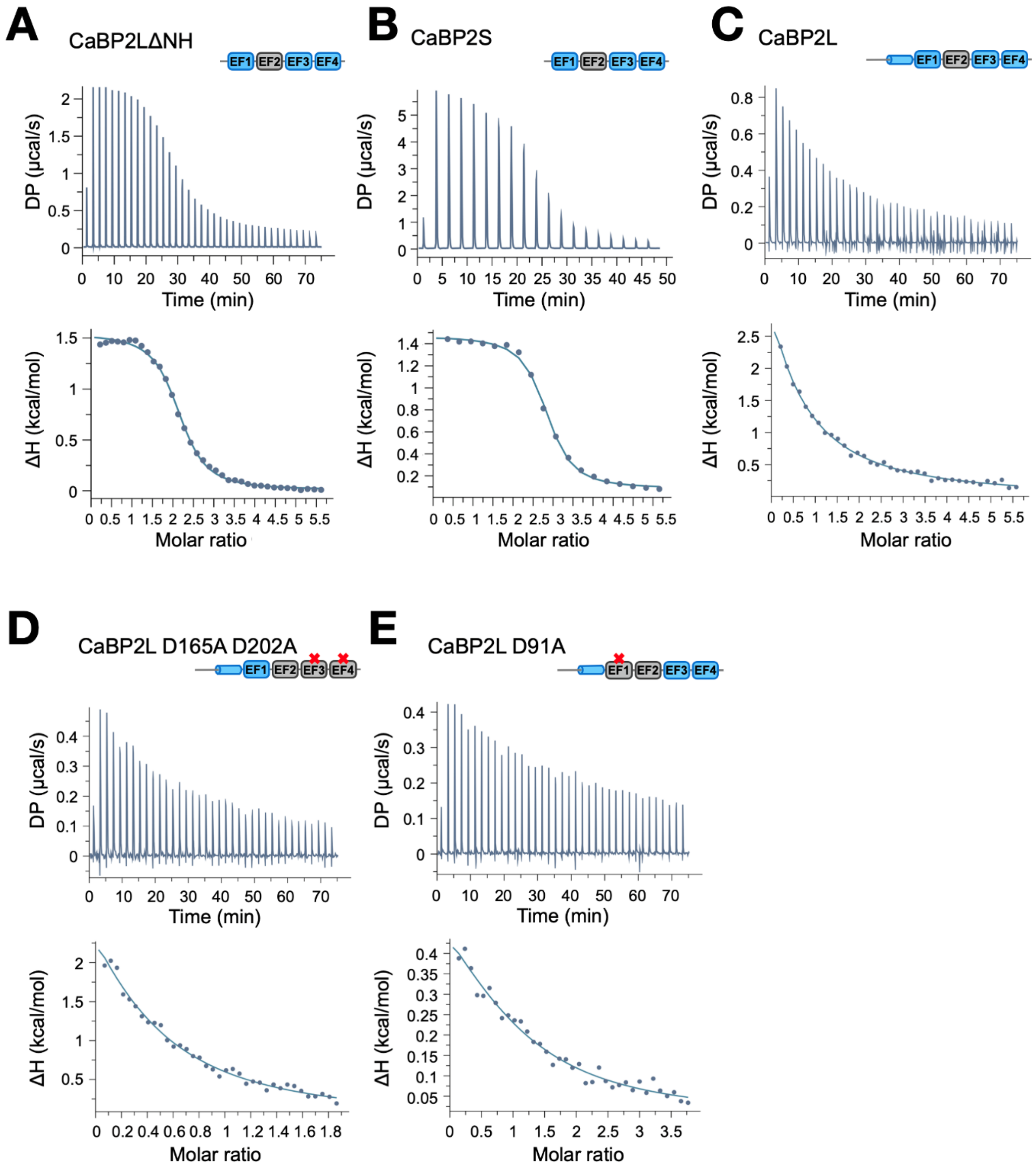

Figure S2 ITC binding assays of CaBP2S and CaBP2L mutants. (A-B) Ca^{2+} binding of CaBP2LΔNH and CaBP2S lacking N-segment and (C-E) Mg^{2+} binding of CaBP2L wild-type and its EF-hand mutants. Thermograms (upper) and binding isotherms (lower) are shown. The quantitative outcomes are presented in Table S1.

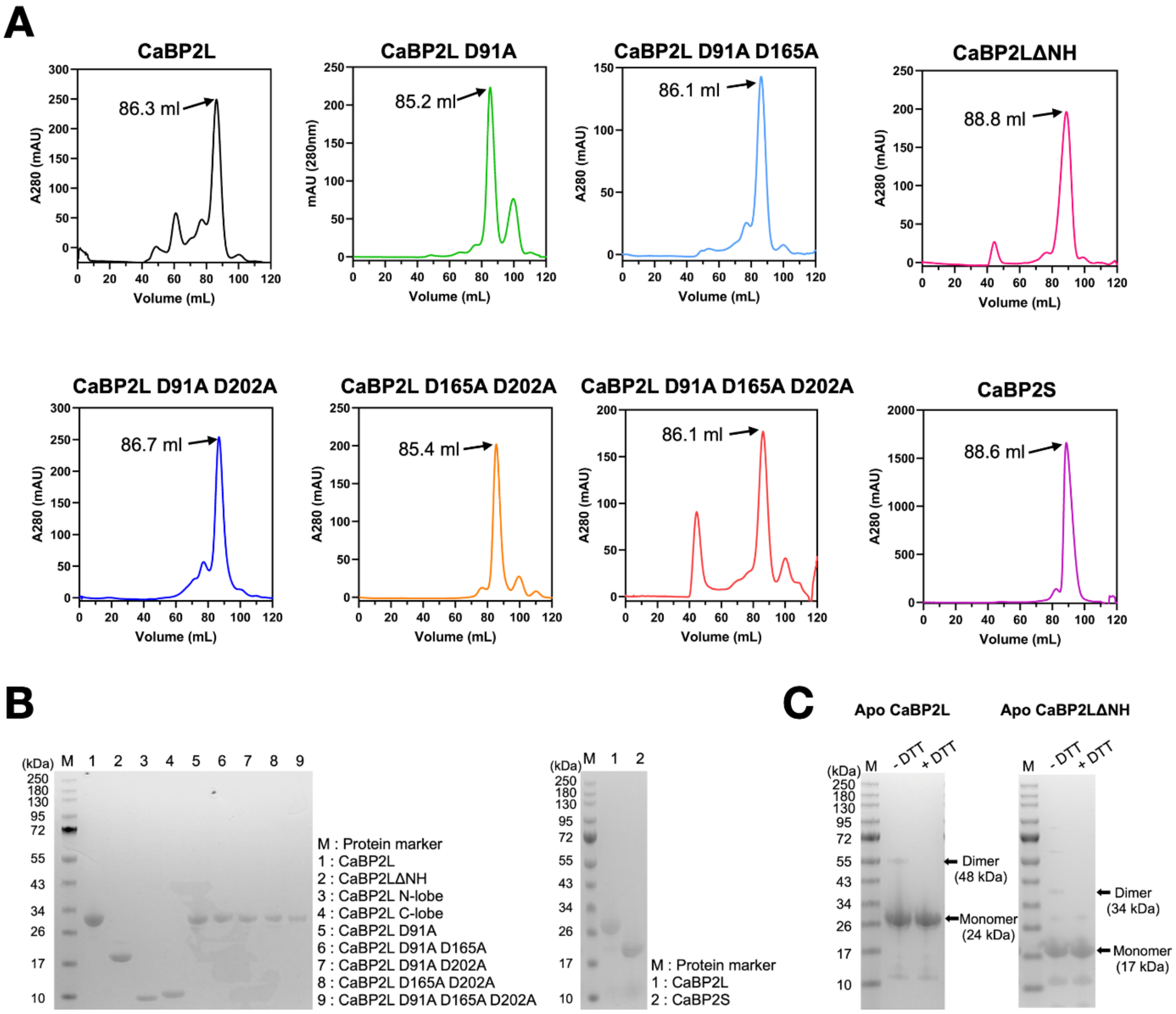

Figure S3 SEC and SDS-PAGE analyses of protein samples used for the biophysical experiments. (A, B) SEC profiles (A) and SDS-PAGE (B) of CaBP2L and its mutants indicate isolation of monomer species. (C) SDS-PAGE analysis of apo CaBP2L and apo CaBP2LΔNH under non-reducing and reducing conditions. Dimeric species observed in the absence of the reducing agent disappeared upon addition of reducing agent dithiothreitol, indicating that the weak dimerization is mediated by intermolecular disulfide bonds.

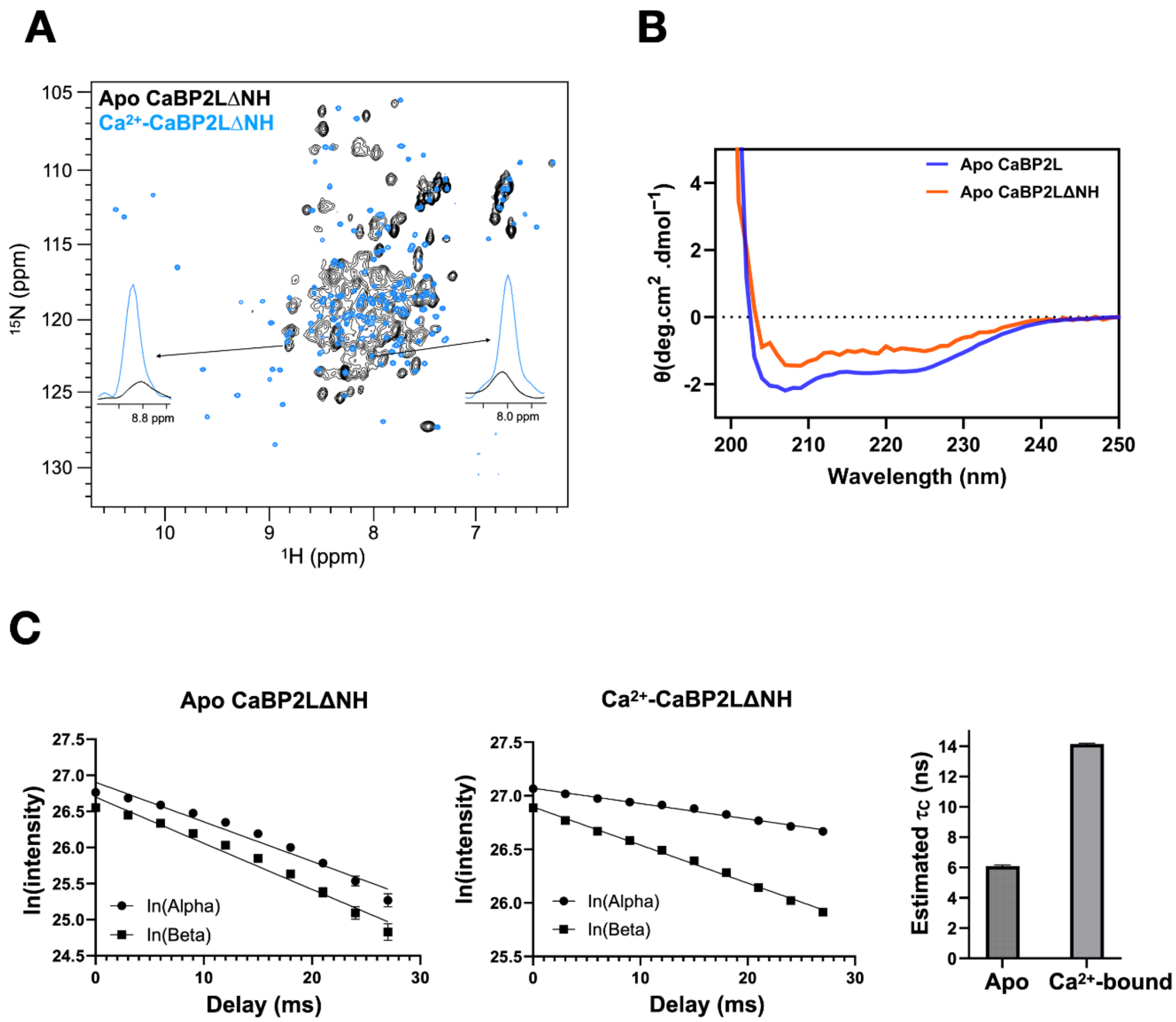

Figure S4 NMR analysis of apo CaBP2LΔNH and Ca^{2+}-CaBP2LΔNH and circular dichroism (CD) spectra of apo CaBP2L and apo CaBP2LΔNH. (A) Comparison of ^{1}H-^{15}N HSQC NMR spectra of ^{13}C, ^{15}N-labeled Apo CaBP2LΔNH (0.4 mM; black) and ^{15}N-labeled Ca^{2+}-CaBP2LΔNH (0.1 mM; blue). Although the protein concentration of the apo CaBP2LΔNH is higher than that of Ca^{2+}-bound CaBP2LΔNH, apo CaBP2LΔNH shows significant line-broadening compared to the Ca^{2+}-saturated CaBP2LΔNH. Peak intensities of two selected resonances, indicated by arrows, are highlighted. (B) CD spectra of CaBP2L and CaBP2LΔNH in the apo state. The secondary structural element of CaBP2LΔNH was not affected in the absence of Ca^{2+} ions. (C) Comparison of rotational correlation times (τ_c) for the apo and Ca^{2+}-bound CaBP2LΔNH determined by TRACT analysis (60), showing an increase in τc upon Ca^{2+} binding. Data are represented as the mean of all analyzed signals and the error values were estimated based on the propagation of spectral noise observed within the integrated amide region. All measurements were performed at 298 K at a magnetic field strength of 600 MHz.

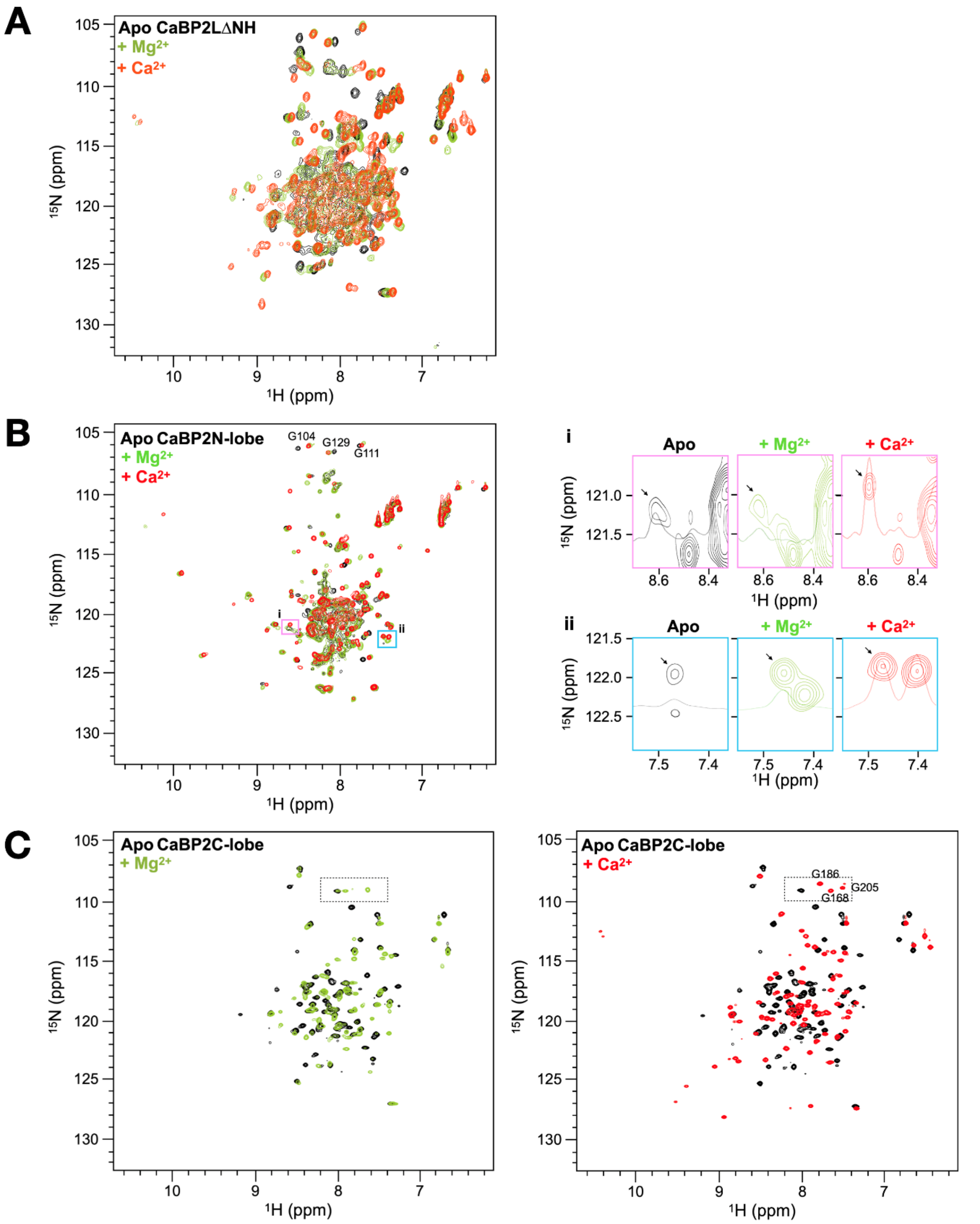

Figure S5 ^1H-^{15}N HSQC NMR spectra of apo CaBP2LΔNH, apo CaBP2L N-lobe, and apo CaBP2L C-lobe in the presence of Mg^{2+} or Ca^{2+}. (A) Overlay of ^1H-^{15}N HSQC NMR spectra of ^{13}C, ^{15}N-labeled CaBP2LΔNH (0.4 mM) in the apo state (black), in the presence of Mg^{2+} (yellow green), and in the presence of Ca^{2+} and Mg^{2+} (orange red). (B) Left; overlay of ^1H-^{15}N HSQC NMR spectra of ^{15}N-labeled apo CaBP2 N-lobe (0.2 mM) in the absence (black), and presence of Mg^{2+} (lime) or Ca^{2+} (red). Resonances of Gly residues (G104, G111, and G129) in the N-lobe were

indicated. Right; zoom-in views of the regions indicated by inset i (pink) and ii (light blue). Relative peak intensities of the apo (black), Mg^{2+}-bound (lime), and Ca^{2+}-bound (red) states are highlighted. (C) Overlay of ^{1}H-^{15}N HSQC NMR spectra of ^{15}N-labeled apo CaBP2L C-lobe (0.2 mM) in the absence (black), presence of Mg^{2+} (left panel, yellow green), and Ca^{2+} (right panel, red). The assigned Gly residues (G168, G186, and G205) in the C-lobe were shown in the spectrum of the Ca^{2+}- bound state.

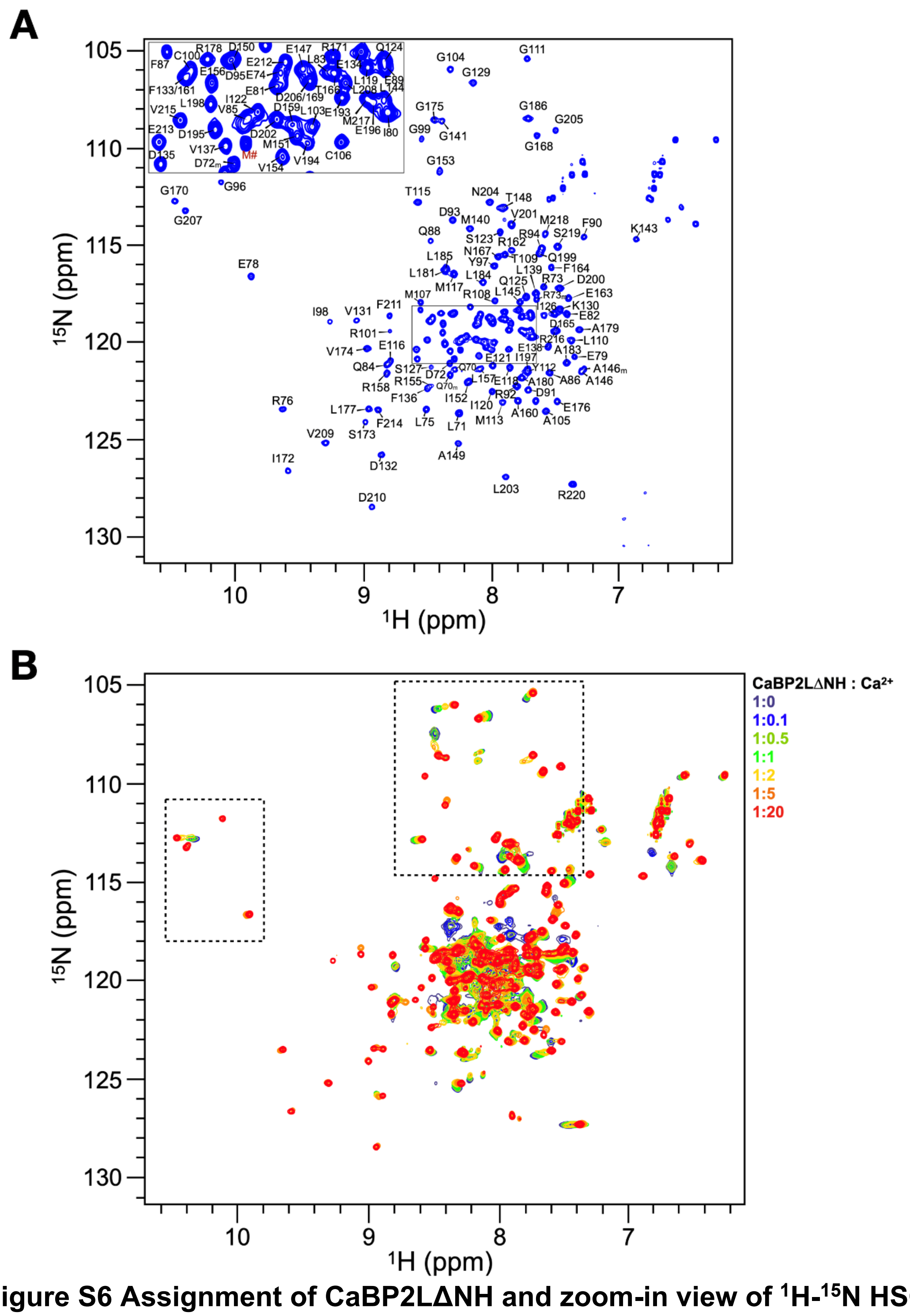

Figure S6 Assignment of CaBP2LΔNH and zoom-in view of ^{1}H-^{15}N HSQC spectra of CaBP2LΔNH at different Ca^{2+} concentrations. (A) ^{1}H-^{15}N HSQC spectrum of CaBP2LΔNH (70–220) at pH 7.5 in the presence of Ca^{2+} (15 mM) recorded at 16.4 T (700 MHz 1H frequency; see the Methods for details). Amino acid residues are labeled in black using their one-letter code. "M#" denotes a plasmid-derived methionine residue. Assignments have been deposited in the BMRB database under accession number 26338. (B) The Spectrum colors correspond to the molar ratios of CaBP2LΔNH and Ca^{2+} shown in the upper right corner. Regions marked with dashed boxes are enlarged in Figure 4C.

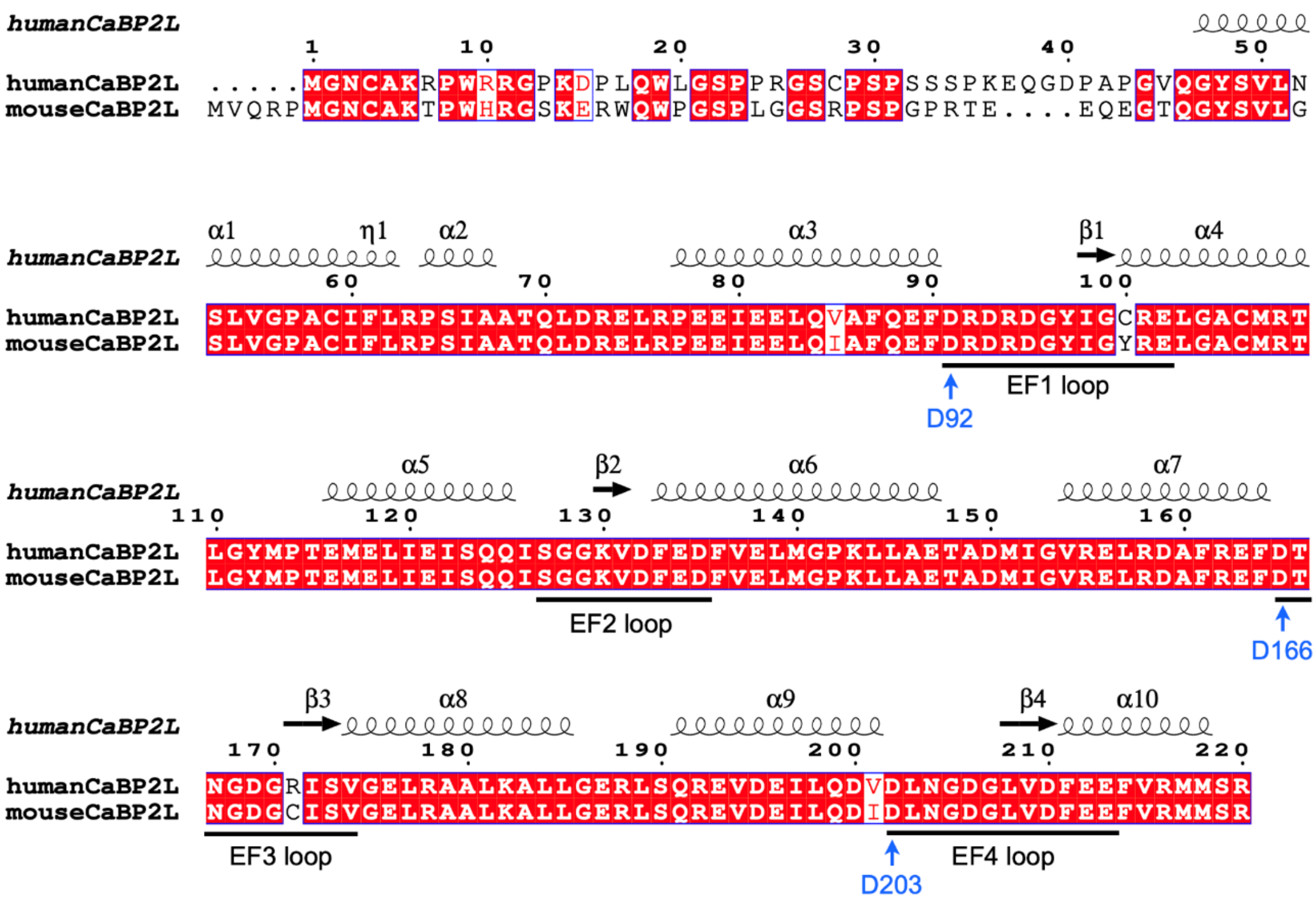

Figure S7 Sequence alignment of human CaBP2L and mouse CaBP2L. Sequence alignment was produced by Clustal Omega (53) and depicted using ESPript 3.2 (54). The UniProt accession numbers of the proteins are as follows: Q9NPB3-1 for human CaBP2L and Q9JLK4-1 for mouse CaBP2L (NP_038906.2). Positions of Asp to Ala mutations used in patch-clamp recordings are highlighted in blue and these correspond to the same locations in human CaBP2L. The predicted secondary structure of the human CaBP2L obtained from AlphaFold (AF-Q9NPB3-F1) (43) is shown on top of the alignment: α, α-helices; β, β-strands; η, 3_{10}-helix.

Table S1. ITC thermodynamic parameters for Ca^{2+} or Mg^{2+} binding to CaBP2S and CaBP2L mutants

Protein	Ligand	K_D (μM)	N (sites)	ΔH (kcal/mol)	-TΔS (kcal/mol)
CaBP2LΔNH	Ca^{2+}	18 ± 5	2.0 ± 0.2	1.60 ± 0.07	-7.7 ± 0.1
CaBP2S	Ca^{2+}	24.0 ± 0.9	2.70 ± 0.02	1.40 ± 0.01	-7.30 ± 0.02
CaBP2L	Mg^{2+}	250 ± 40*	N.D.	N.D.	N.D.
CaBP2L D165A D202A (EF1 active)	Mg^{2+}	160 ± 27	0.37 ± 0.06	6.2 ± 1.1	-11 ± 1
CaBP2L D91A (C-lobe active)	Mg^{2+}	210 ± 99	0.84 ± 0.31	1.2 ± 0.9	-6.1 ± 0.4

N.D. stands for not determined by ITC. Data are presented as mean ± SD from independent experiments.

* Apparent dissociation constant determined by ITC using a one-site binding model.

Table S2. List of primers used to create CaBP2L mutants

Primer name	Sequence (5'→3')
CaBP2_D91A_F	GAGTTTGCCCGTGATCGTGACGGTTA
CaBP2_D91A_R	ATCACGGGCAAACTCTTGGAACGCAA
CaBP2_D165A_F	GAATTTGCTACCAACGGTGACGGCCG
CaBP2_D165A_R	GTTGGTAGCAAATTCACGGAACGCGTC
CaBP2_D202A_F	GACGTGGCCCTGAATGGCGATGGCCTG
CaBP2_D202A_R	ATTCAGGGCCACGTCTTGCAGAATTTC
CaBP2_delN_F	CAGCTGGACCGTGAACTGCG
CaBP2_delN_R	CATATGGCTGCCGCGCGGCA
CaBP2_Nlobe_NheI_F	AAACATATGCAGCTGGACCGTGAACTG
CaBP2_Nlobe_BamHI_R	AAAGGATCCTTAGATCATATCCGCGGTTTC
CaBP2_Clobe_NheI_F	AAACATATGGATATGATCGGCGTTCG
CaBP2_Clobe_BamHI_R	AAAGGATCCTTAGCGGCTCATCA

Table S3. Sample concentration for ITC experiments

Syringe	Cell	[Syringe]/M	[Cell]/M
Ca^{2+}	CaBP2L	6.43E-03	2.14E-04
Ca^{2+}	CaBP2L D165A D202A	2.46E-03	2.46E-04
Ca^{2+}	CaBP2L D91A D202A	2.16E-03	2.16E-04
Ca^{2+}	CaBP2L D91A D165A	2.19E-03	2.15E-04
Ca^{2+}	CaBP2L D91A	4.82E-03	2.41E-04
Ca^{2+}	CaBP2L D91A D165A D202A	3.78E-03	3.78E-06
Ca^{2+}	CaBP2LΔNH	7.90E-03	2.65E-04
Ca^{2+}	CaBP2S	1.89E-02	6.30E-04
Mg^{2+}	CaBP2L	4.50E-03	1.50E-04
Mg^{2+}	CaBP2L D165A D202A	2.50E-03	2.53E-04
Mg^{2+}	CaBP2L D91A	4.95E-03	2.48E-04
